# Comparative analysis of simulated *in-situ* colonization and degradation by *Lentinula edodes* on oak wafer and corn stalk

**DOI:** 10.3389/fmicb.2023.1286064

**Published:** 2023-11-23

**Authors:** Chunye Mou, Yuhua Gong, Lianfu Chen, Francis Martin, Heng Kang, Yinbing Bian

**Affiliations:** ^1^College of Plant Science and Technology, Huazhong Agricultural University, Wuhan, China; ^2^Hubei Hongshan Laboratory, Wuhan, China; ^3^Université de Lorraine, INRAE, UMR Interactions Arbres/Microorganismes, Centre INRAE Grand Est-Nancy, Champenoux, France

**Keywords:** white-rot fungus, lignocellulose degradation, carbohydrate-binding module, transcriptome, compositional change, scanning electron microscopy

## Abstract

**Introduction:**

The depolymerization of lignocellulose biomass by white-rot fungi has been an important research topic. However, few simulated in-situ analyses have been conducted to uncover the decay.

**Methods:**

In this study, the white-rot Lentinula edodes was used to colonize the wood and non-wood substrates, and then hyphal transcriptional response and substrate degradation were analyzed during the spatial-temporal colonization on different type substrates to better understand the depolymerization of lignocellulose.

**Results and discussion:**

Faster growth and thicker mat of hyphae on corn stalk were observed in comparison to oak wafer. Coincide with the higher levels of gene transcripts related to protein synthesis on corn stalk. The higher lignin oxidase activity of hyphae was detected on oak wafer, and the higher cellulase activity was observed on corn stalk containing a much higher content of soluble sugars. A large number of carbohydrate-binding module (CBM1 and CBM20)-containing enzyme genes, including lytic polysaccharide monooxygenase (AA9), cellobiohydrolase (GH6 and GH7), glucanase (GH5), xylanase (GH10 and GH11), glucoamylase (GH15), and alpha-amylase (GH13), were significantly upregulated in the back-distal hyphae colonized on corn stalk. The hyphae tended to colonize and degrade the secondary cell wall, and the deposited oxalate crystal suggested that oxalate may play an important role during lignocellulose degradation. In addition, lignin was degraded in priority in oak wafer. Of note, three lignin monomers were degraded simultaneously in oak wafer but sequentially in corn stalk. This growth Our results indicated that the white-rot degradation pattern of lignocellulose is determined by the chemical composition and structure of the colonized biomass.

## Introduction

Lignocellulose is the major component of the plant cell wall (PCW) and it has been regarded as a large carbon (C) resource on the earth with promising potential to be used in the production of edible mushroom and value-added products (Philippoussis, 2009; Chen, 2014; Tang et al., 2019). However, PCW generally has a low enzymatic digestibility due to its structural complexity and the recalcitrance of lignin-related compounds (Himmel et al., 2007; Martínez et al., 2009). Usually, PCW consists of primary and secondary cell walls, and secondary cell wall include three main layers, with the cellulose-rich S2 layer being sandwiched by lignified S1 and S3 layers (Watkinson, 2016). Hydrogen bonds link unbranched or less branched hemicelluloses to cellulose fibrils, while the hemicellulose side chains are covalently bound to lignin, forming an enzyme-impenetrable cross-linking network (Xie and Peng, 2014). Moreover, lignin, which is composed of three phenylpropanoid monomers (H, p-hydroxyphenyl; G, guaiacyl; S, syringyl) establishing several types of covalent bonds, is a hydrophobic phenolic heteropolymer, highly recalcitrant to microbial decomposition (Ralph et al., 2004; Watkinson, 2016).

In recent years, lignocellulose degradation has been widely studied using -omics technologies, which has significantly improved our understanding of PCW degradation. For example, to obtain nutrients from extracellular carbohydrates, enzyme proteins must be secreted in accordance with the available carbon source (Brown et al., 2014). Several reports have indicated that fungal growth in solid-substrate fermentation is quite different from that in liquid medium, resulting in different patterns of enzyme production (Datta et al., 1991; Dawson-Andoh and Morrell, 1992; Daniel, 1994; Zhao et al., 1996; Daly et al., 2018). In addition, environmental features experienced by degrading hyphae in submerged mycelium cultures or growing on powdered wood under laboratory conditions are quite different from those taking place in natural environments, which may mask some key responses of fungal hyphae (Daly et al., 2018). Generally, the availability, quality and complexity of C sources affect the secretome composition (McCotter et al., 2016). Besides, different patterns of growth and enzyme secretion have been reported in *Serpula lacrymans* and *Gloeophyllum trabeum* with significant taxonomic and niche distances (Presley and Schilling, 2017). In addition, the activity of enzymes related to lignin degradation is also affected by bio-available nitrogen (N) and C levels (Mikiashvili et al., 2006). Therefore, proper N supplementation in the growth medium has been identified as one of the key factor for hyphal production and substrate conversion (Bellettini et al., 2019). A spatial–temporal method of hyphal colonization has been used in brown-rot and white-rot fungi and revealed the staggered spatial expression of oxidation- and hydrolysis- related genes of Rhodonia (*Postia*) *placenta* (Zhang et al., 2016). However, little research has been carried out to mimic and reflect the decomposition of solid-state natural substrates by white-rot fungi. Therefore, it is critical to uncover the mechanisms of lignocellulose depolymerization, especially the degradation that takes place in the environments close to nature, by white-rot fungi to improve the understanding of the bio-conversion for lignocellulosic biomass.

White-rot fungi are among the most efficient microorganisms to degrade cellulose and hemicellulose (Martínez et al., 2005; Fernandez-Fueyo et al., 2012). In addition, they have also evolved a prominent capability of efficiently depolymerizing and mineralizing lignin, which is the most recalcitrant component in PCW (Martínez et al., 2005; Fernandez-Fueyo et al., 2012; Floudas et al., 2012). The degradation of lignin and polysaccharides by white-rot fungi mainly relies on the activity of oxidoreductases and glycoside hydrolases, respectively (Sánchez, 2009; Floudas et al., 2012; Qin et al., 2018; Pastorelli et al., 2020). The white-rot fungus *Lentinula edodes*, also called as Xianggu in China or shiitake in Japan, produces a large suit of extracellular enzymes to degrade cellulose, hemicellulose, and lignin (Chen et al., 2016). It is one of the most important commercially cultivated edible mushrooms around the world (Royse et al., 2017; Baktemur et al., 2020) and is mainly cultivated with sawdust-based medium (Cai et al., 2017). The wild strains have a strong preference for Fagaceae (Menolli et al., 2022). Recently, corn stalk, which contains higher levels of bio-available C and N than sawdust, has been tentatively used, partially, as a cultivation substrate for mushroom production (Atila, 2019; Chen et al., 2021). There is a massive production of corn straw during the corn production every year, and it represents a huge potential for biomass utilization and mushroom production (Li H. et al., 2017; Mohd Hanafi et al., 2018; Meng et al., 2019). As a common white-rot fungus and edible mushroom, extensive researches in the cultivation, including issues related to genetic mapping of the agronomic trait (Li C. et al., 2017), response to heat stress (Wang et al., 2020; Xu et al., 2022), formation of brown film (Tang et al., 2022) and substrate degradation (Huang et al., 2020), have been conducted. So, it showed great practical application value to explore the depolymerization of lignocellulose using *L*. *edodes*.

In this study, we conducted an *in vitro* vertically spatial–temporal growth to mimic *in-situ* colonization and degradation and followed the gene expression and changes in physico-chemical characteristics of the substrates to better understand lignocellulose degradation. Oak wood wafers (ow) and corn stalk slices (cs) were simultaneously incubated in the presence of *L. edodes* hyphae. Then, transcriptome profiling and enzyme assays were carried out to characterize the spatial and temporal patterns of expression of the enzymes involved in lignocellulose degradation. Scanning electron microscopy and substrate chemical assays were performed to assess the changes in the substrate composition and structure.

## Materials and methods

### Colonization of substrates by *Lentinula edodes* hyphae

**Figure 1:**
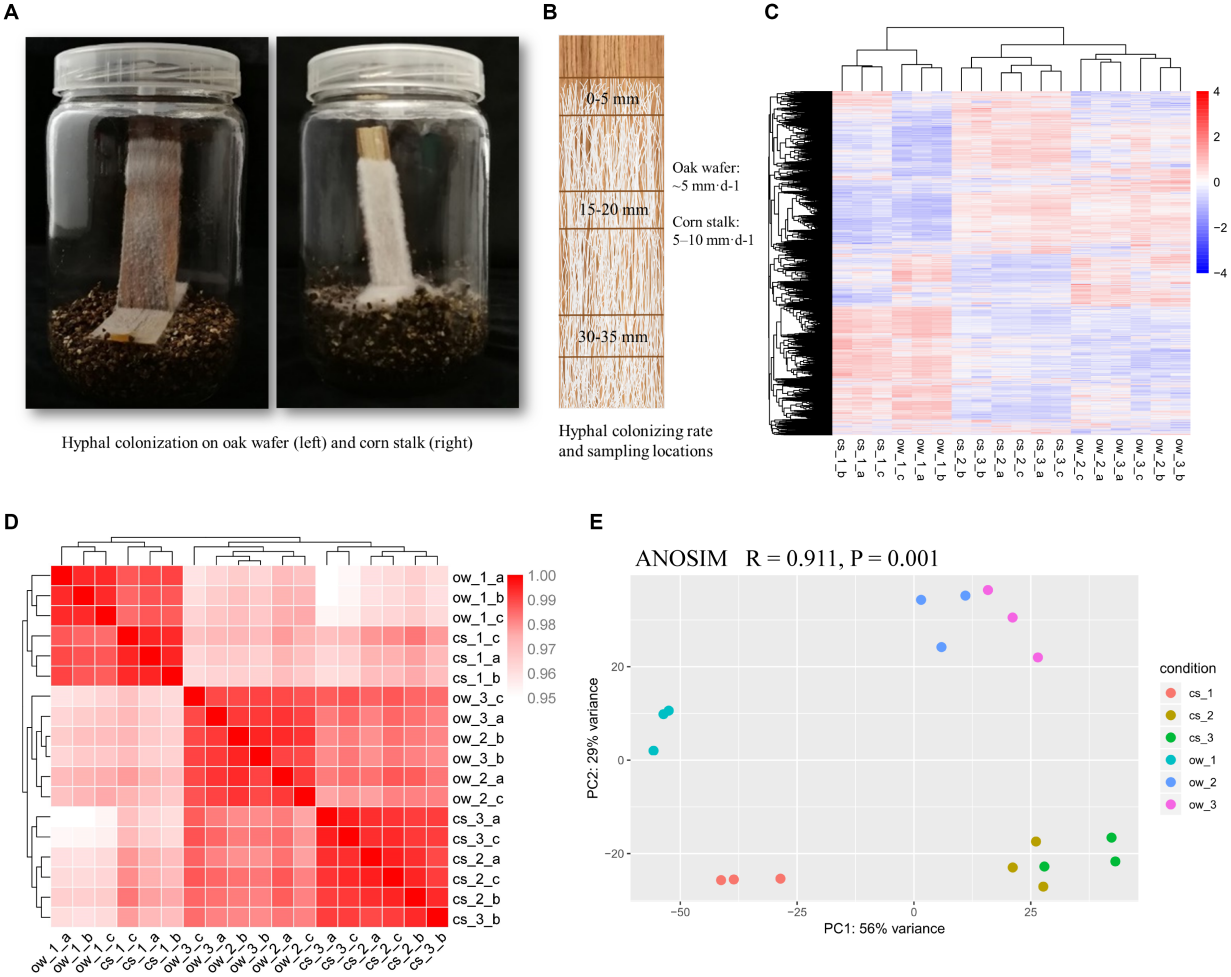
Hyphal colonization and transcriptome similarity of *L. edodes* on oak wafer and corn stalk. **(A)** Hyphal colonization on substrate wafer (left, oak wafers; right, corn stalk). **(B)** The schematic diagram shows the locations of sampling section starting at the advancing colonization front and the colonizing rate of hyphae. **(C)** Hierarchical clustering analysis of spatial–temporal gene expression along the advancing hyphae. **(D)** Pearson correlation coefficient-based heatmaps and **(E)** principal component analyses (PCA) showing the similarity of available gene expression among various colonization stages on oak wafer and corn stalk. The dark red color in B indicates higher similarity. Ow, oak wafer; cs, corn stalk. Section locations for both substrates: 0–5 mm (section 1), ow_1 or cs_1; 15–20 mm (section 2), ow_2 or cs_2; 30–35 mm (section 3), ow_3 or cs_3. Biological replicates are indicated using a-c.

Substrate colonization and sampling were conducted according to a previous study (Zhang et al., 2016) with some minor modifications (Figure 1A; see Supplementary Information). From the visible hyphal-colonization front, the substrates covered with mycelia were cut into 5-mm sections along the hyphal colonization direction at 10-mm intervals, and three locations were sampled in total (four locations were selected for four main substrate components assay). The samples of hyphae and substrate sections were used for the following RNA-Seq, extracellular enzyme activity assays, substrate degradation observation, and composition determination. Distance from the hyphal colonization-front was used to describe the sections on substrates.

### Total RNA extraction, RNA-sequencing and data analysis

The samples of *L. edodes* hyphae grown on oak wafers and corn stalks were snap-frozen in liquid N_2_ stored at – 80°C, and then sent to BGI Genomics (Shenzhen, China) for RNA extraction and sequencing. Three biological replicates were performed. The extraction of total RNA and the examination of concentration, quality, and purity were performed following the manufacturer’s protocol from BGI Genomics. Then, the construction of eighteen cDNA libraries and RNA-Seq sequencing were performed at BGI Genomics according to their standard Illumina protocols. The 150-bp long paired-end reads were generated on the Illumina HiSeq X-ten sequencer. The Analysis of similarity (ANOSIM), a non-parametric multivariate statistical test, was performed to test the difference among the spatial–temporal groups on different substrates using package vegan. The differential expression analysis was performed using DESeq2 (Love et al., 2014). Genes with a fold change ≥2 and a false discovery rate (FDR) <0.01 were designated as differentially expressed genes (DEGs). DEGs were functionally annotated by blast search against the databases of Pfam, Swiss-Prot, NR, KEGG, GO, KOG, Interpro, carbohydrate-active enzymes (CAZymes) as previously described (Chen et al., 2016). The expression of several enzyme genes related to lignocellulose degradation was tested by qRT-PCR along the colonization process on substrates according to a previous study (Cai et al., 2017) and the gene-specific primers are shown in Supplementary Table S1. The RNA-seq data reported in this paper have been deposited in the Genome Sequence Archive (Chen et al., 2021) in the National Genomics Data Center (Members, CNCB-NGDC, 2022) (GSA: CRA010424).

### Enzyme activity assays

Every hyphal sample on sections of substrates was split with one half being soaked in 0.05 M citrate buffer (pH 5.0) for 24 h at 4°C to extract extracellular lignocellulolytic enzymes and the other half being used to approximate the biomass of *L. edodes* hyphae for each section. In total, eight corn stalk and fifteen oak wafer sections were pooled for each sample location. Endoglucanase (CMCase), xylanase, manganese peroxidase (MnP), and laccase activities were assayed according to previous methods (Zhang et al., 2016; Cai et al., 2017) with minor modifications. NaOH-extractable proteins were measured as described by a previous study (Zhang et al., 2016) and protein concentration was determined using the Bradford Protein Assay Kit (Beyotime, P0006). All enzyme activities were normalized by fungal biomass.

### Scanning electron microscopy (SEM) of substrate decay

For SEM, sections of the substrates were cut into fragments <5 × 5 mm and soaked into glutaraldehyde for more than 3 h, followed by dehydration with an increasing series of ethanol (from 20 to 98%, v/v). Well-dried sections (colonized by *L. edodes* hyphae) were sputter-coated with gold in a JFC-1600 ion sputter (Mito City, Japan). To assess the impact of the hyphal colonization, the substrate surface was scanned by using SEM JSM-6390/LV (Hitachi, Tokyo, Japan). Five to eight images were saved for each sample to acquire representative images.

### Hemicellulose and cellulose extraction

For lignocellulose component analysis, the samples were hot-air dried, ground into powder, and then passed through a 40 mesh sieve. Extraction of hemicellulose and cellulose from the PCW of each substrate section was performed as previously described (Peng et al., 2000; Wu et al., 2013). The cellulose content was estimated by calculating the hexoses from the non-KOH-extractable pellet using the anthrone/H_2_SO_4_ method (Fry, 1988). D-glucose and D-xylose (Sinopharm Chemical Reagent Co., Ltd.) were prepared to plot standard curves of hexose, pentose, and uronic acids, respectively. Considering the interference of pentose on the hexose reading at 620 nm, the pentose OD reading at 660 nm was deducted for the calculation of hexose. All experiments were conducted in independent triplicates.

### Content determination of lignin and three monomers

The ground powder of the substrate section was also used for lignin content analysis. Total lignin content of the samples, including acid insoluble lignin (AIL) and acid-soluble lignin (ASL), was determined using the two-step acid hydrolysis method according to the Laboratory Analytical Procedure of the National Renewable Energy Laboratory (Sluiter et al., 2008). AIL was calculated gravimetrically after correction for ash, whereas ASL was measured using UV spectroscopy as previously described (Zhang et al., 2021). Lignin monomers were extracted through Nitrobenzene Oxidation as described in a previous study (Li et al., 2014b). Three lignin monomers (H-, G-, and S-) were analyzed by the HPLC method (1,525, Waters Corp., MA, United States) (Wu et al., 2013; Li et al., 2014b). All analyses were performed in independent triplicates.

## Results

### Colonization of the substrate by *Lentinula edodes* hyphae

*L. edodes* mycelial cultures were grown on vertical oak wood wafers and corn stalk slices without additional nutrients (Figure 1A). A thicker hyphal lawn and higher extension rate was shown on corn stalk slices than on oak wafer. The mycelial colonization rate was ~5 mm·d^−1^ and 5–10 mm·d^−1^ on oak wafer and corn stalk, respectively. A section of 0–5 mm was sampled at the advancing hyphal-colonization front (section 1) to represent the initial substrate colonization stage, and the following sections – 2 (15–20 mm) and 3 (30–35 mm) – were taken at an interval of 10 mm to assess the advanced degradation process (Figure 1B). A distinct temporal and spatial expression patterns of several lignocellulose degradation-related genes in hyphae colonizing oak wafers and corn stalk slices was estimated by qPCR assays before RNA-Seq profiling (Supplementary Figure S1).

### Spatial–temporal transcriptional profiling on oak wafers and corn stalk slices

Based on the results of hyphal growth and gene expression related to lignocellulolytic enzymes, three above-described sections, covered by mycelium, were taken to perform the transcriptome analysis. A total of 11,482 transcripts were mapped to the reference genome, accounting for 77% of the total predicted protein-coding genes (number, 14889) (Supplementary Data S1) (Chen et al., 2016). The gene expression profiles of hyphae from sections 2 and 3 clustered and were separated from section 1 profile, indicating that the gene expression pattern was similar during the late stages of the colonization process on both substrates (Figures 1C,D). PC1 and PC2 of the principal component analysis (PCA) explained 85% of the total variance. In addition, PC1 separated section 1 from sections 2 and 3 on both substrates, and PC2 indicated a difference in degradation response between the two substrates (Figure 1E). In general, transcriptome profiles of hyphae colonizing oak wafers and corn stalk slices were significantly distinct (ANOSIM, R = 0.911, *p* = 0.001) for the three temporal–spatial sections (Supplementary Figure S2). These results revealed that the hyphae served different functions at different spatial–temporal locations of colonization and also revealed that the different substrates might have initiated different responses of degradation.

### DEGs from hyphal-colonization front to backend on oak wafer and corn stalk

DEGs were identified by pairwise comparisons (ow_2 *vs* ow_1, ow_3 *vs* ow_1, ow_3 *vs* ow_2, cs_2 *vs* cs_1, cs_3 *vs* cs_1, cs_3 *vs* cs_2, ow_1 *vs* cs_1, ow_2 *vs* cs_2, ow_3 *vs* cs_3) over the colonization of oak wafers and corn stalk slices or between different substrates. In total, 3,845 (2,304 up, 1,541 down), 4,355 (2,628 up, 1727 down), 127 (111 up, 16 down), 3,399 (2018 up, 1,381 down), 4,054 (2,360 up, 1,694 down), 55 (22 up, 33 down), 1,072 (481 up, 591 down), 1,562 (827 up, 735 down), 1,147 (666 up, 481 down) DEGs were identified (Supplementary Figure S3; Supplementary Data S2). In particular, a higher fold change of gene expression was identified in hyphae colonizing on oak wafer. In short, the expression of 32% (Percentage of DEG numbers against total predicted gene numbers, 4,724:14889) or 29% (4,318:14889) of the *L. edodes* predicted genes was significantly altered when grown on oak wafers or corn stalk slices, respectively (Supplementary Data S2).

On both substrates, DEGs significantly upregulated in section 1 were mainly related to synthesis, energy metabolism, and transmembrane transport (Supplementary Data S3). We also identified terms related to oxidoreductase activity in section 1, such as heme acting on the donors (GO:0016675), indicating that the oxidative degradation of lignin was already initiated in the hyphal-colonization front. However, numerous genes related to oxidoreductase activity (GO:0016491) and hydrolase activity (GO:0016787) were significantly enriched in section 2. In section 3, genes related to the hydrolase activity, such as those acting on glycosyl bonds (GO:0016798), were significantly enriched, indicating a progressive degradation stage and synchronization of oxidation and hydrolysis process, especially, on oak wafer.

By comparison, fewer DEG were identified at the same spatial–temporal position between different substrates (0–5 mm: ow_1 *vs* cs_1, 15–20 mm: ow_2 *vs* cs_2 and 30–35 mm: ow_3 *vs* cs_3) (Supplementary Figure S3; Supplementary Data S2). For these three comparisons, the GO enrichment analysis of DEGs showed greater differences in GO terms from the early to later colonization stages (Supplementary Data S3). In section 1, the terms, that is carboxylic acid transmembrane transporter activity (GO:00046943) and calcium ion binding (GO:0005509), were found on oak wafers. However, on corn stalk slices, GO terms of monooxygenase/dioxygenase activity (GO:0004497/GO:0051213) and transition metal ion binding (GO:0046914) were identified. In contrast, sections 2 and 3 exhibited more advanced degradation. Some additional terms of monooxygenase activity (GO:0004497), and hydrolase activity (GO:0016810), acting on carbon-nitrogen (but not peptide) bonds were significantly enriched on oak wafers, while hydrolase activity (GO:0016787), cellulose binding (GO:0030248) were significantly enriched on corn stalk slices. In section 3 (30–35 mm), enriched peroxidase activity (GO:0004601) indicated a sustained lignin degradation on oak wafers.

### Spatial–temporal expression of genes related to lignocellulose degradation

**Figure 2:**
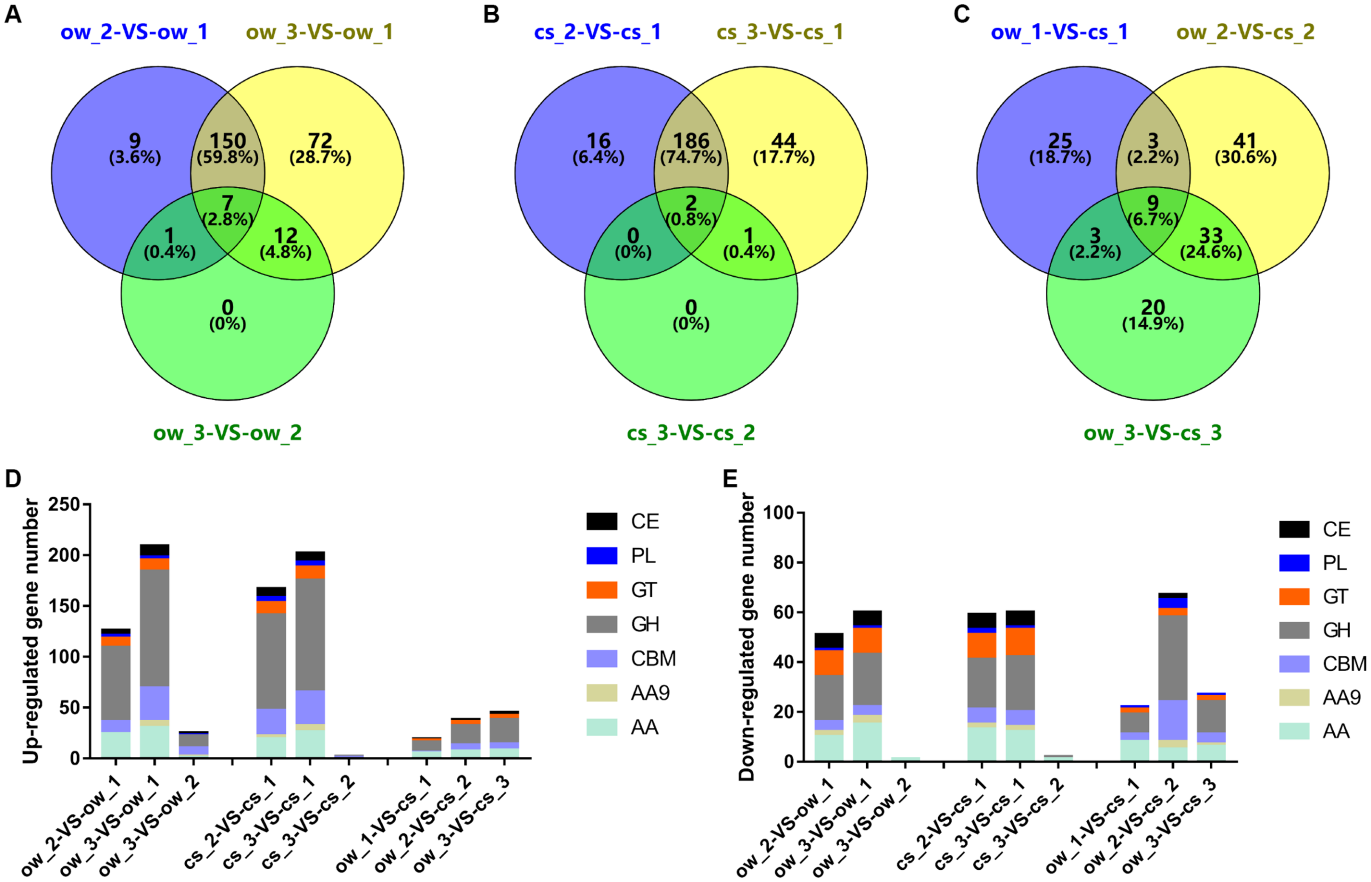
Statistics of CAZyme-encoding DEGs. **(A–C)** Venn diagram shows the DEG number of CAZyme between the temporal–spatial sections of the same substrate and the same locational sections of the different substrates. **(D,E)** The bar chart shows the gene distribution of CAZyme families (GH, AA, AA9, CBM, GH, GT, PL, CE). Section locations for both substrates: 0–5 mm (section 1), ow_1 or cs_1; 15–20 mm (section 2), ow_2 or cs_2; 30–35 mm (section 3), ow_3 or cs_3.

The genome of the *L. edodes* monokaryon strain W1-26 (derived from strain W1) encodes 456 CAZyme genes (Chen et al., 2016). The largest number of DEGs coding for CAZymes was identified between section 1 and section 2 or section 3 on both substrates (Figures 2A–C). They mainly code for glycoside hydrolases (GHs, 115 genes in ow_3 *vs* ow_1), followed by auxiliary activities (AAs, 36 genes in ow_3 *vs* ow_1) (Figures 2D,E). In addition, the most DEGs (gene number 33) of enzyme containing carbohydrate-binding modules (CBMs) were identified in cs_3 *vs* cs_1.

A minor set of genes coding for lignocellulose degradation enzymes (LDEs) was highly induced in section 1 on both substrates (Table 1; Supplementary Figures S4–S6; Supplementary Data S4). Among the lignin modification-related enzyme, one peroxidase (AA2, LE01Gene03920), two aryl-alcohol oxidases (AA3_2a, LE01Gene09147 and LE01Gene09156), one copper radical oxidase (AA5_2, LE01Gene12661) and two glucooligosaccharide oxidase (AA7, LE01Gene02931 and LE01Gene09065) genes were significantly upregulated. Besides, the expression of a few genes related to plant cell wall polysaccharides degradation also significantly upregulated, such as lytic polysaccharide monooxygenase (LPMO, AA9, LE01Gene03338 and LE01Gene14047), endo-beta-1,4-glucanase (GH5_5-CBM1, LE01Gene12259; GH9, LE01Gene08227), glucan 1,3-beta-glucosidase (GH5_9, LE01Gene11040), alpha amylase (GH13, LE01Gene12213, LE01Gene13578, LE01Gene01055), chitin deacetylase (CE4, LE01Gene01633, LE01Gene03495, LE01Gene11812). In particular, the glucooligosaccharide oxidase (AA7, LE01Gene13975), glucose oxidase (AA3_2, LE01Gene12439), chitin deacetylase (CE4, LE01Gene02924) were exclusively upregulated in section 1 of corn stalk. And the laccase (AA1, lac5, LE01Gene01372; lac14, LE01Gene04660), LPMO (AA9, LE01Gene08464) and alpha amylase (GH13, LE01Gene03470) were upregulated in section 1 of oak wafer. This suggests that lignocellulose degradation was initiated from section 1 (0–5 mm), and showed a different pattern, to a certain extent, responding to two substrates.

**Table 1:** Summary of lignocellulolytic DEGs during the spatial–temporal colonization on oak wafer and corn stalk slice.

Family	Putative Function	SP^a^	Gene ID^b^	Fold-changes for different comparisons								
				ow2 *vs* ow1	ow3 *vs* ow1	ow3 *vs* ow2	cs2 *vs* cs1	cs3 *vs* cs1	cs3 *vs* cs2	ow1 *vs* cs1	ow2 *vs* cs2	ow3 *vs* cs3
AA1	Laccase 8	Y	01361	1.4	1.4	–	–	–	–	−1.5	–	–
AA1	Laccase 5	Y	01372	−4.5	−4.1	–	–	–	–	–	−3.6	−3.8
AA1	Laccase 9	Y	04648	–	–	–	1.2	1.7	–	2.0	--	--
AA1	Laccase 14	Y	04660	–	−5.3	−5.8	–	–	–	5.0	--	--
AA1	Laccase 2	Y	07443	–	1.0	–	--	1.0	–	--	--	--
AA1	Laccase 7	Y	07758	–	−1.4	–	−1.4	−1.7	–	--	--	--
AA1	Laccase 1	Y	08330	2.3	2.7	–	1.4	1.9	–	−1.5	--	--
AA1	Laccase 6	Y	13,044	–	--	–	5.7	7.4	–	--	−3.4	−5.3
AA1	Laccase 10	N	05032	–	1.6	–	1.1	1.3	–	−1.6	−1.8	−1.3
AA1	Laccase 11	N	06108	1.0	1.5	–	1.3	1.3	–	--	–	–
AA2	Manganese peroxidase	Y	09060	2.4	3.9	–	–	–	–	–	–	2.5
AA2	Other peroxidase	N	03530	1.3	1.9	–	–	–	–	−1.0	–	–
AA2	Other peroxidase	N	03920	−3.2	−4.0	–	−2.9	−2.9	–	–	–	–
AA2	Manganese peroxidase	N	08997	4.7	7.0	–	5.0	4.3	–	–	–	3.4
AA2	Versatile peroxidase	N	10,965	–	1.2	–	–	–	–	–	–	–
AA3	Glucose oxidase	Y	07621	4.8	6.3	–	3.6	3.9	--	--	--	--
AA3	Glucose oxidase	Y	12,439	–	–	–	−1.2	−1.3	–	–	–	–
AA3	Aryl-alcohol oxidase	N	04820	4.3	5.0	–	3.5	4.0	–	−1.6	–	–
AA3	Aryl-alcohol oxidase	N	04836	2.8	4.8	2.0	3.7	5.1	1.4	–	−1.6	−1.1
AA3	Aryl-alcohol oxidase	N	09147	−3.6	−4.1	–	−2.9	−2.7	--	1.4	–	–
AA3	Alcohol oxidase	N	09192	−2.7	−3.1	–	−2.6	–	–	–	–	–
AA3	Alcohol oxidase	N	10,868	2.9	3.4	–	–	–	–	–	3.1	2.9
AA3;AA8	Aryl-alcohol oxidase	Y	09156	−3.3	−3.4	–	−2.2	−2.1	–	1.4	–	–
AA3;AA8	Aryl-alcohol oxidase	N	04010	1.7	2.1	–	1.3	1.8	–	–	–	–
AA3;AA8	Cellobiose dehydrogenase	N	04232	–	3.2	–	--	4.8	–	–	–	–
AA3_4	Pyranose oxidase	N	02678	3.9	4.4	–	5.1	4.8	–	–	–	–
AA5	Glyoxal oxidase	Y	04793	--	1.9	–	1.2	1.7	–	–	–	–
AA5	Glyoxal oxidase	N	02092	4.7	6.8	–	1.7	2.2	--	−2.4	--	2.2
AA5	Glyoxal oxidase	N	04633	–	–	–	−1.0	--	–	–	–	–
AA5	Galactose oxidase	N	12,661	−1.6	−1.6	–	−1.6	−1.7	–	–	–	–
AA7	GOOX	N	00015	1.6	3.0	–	1.7	–	–	–	–	1.8
AA7	GOOX	N	00047	1.1	1.2	–	--	1.1	–	–	–	–
AA7	GOOX	N	02931	−2.9	−3.2	–	−1.9	−2.1	–	1.7	–	–
AA7	GOOX	N	05597	3.1	2.2	–	--	2.6	–	–	2.0	–
AA7	GOOX	N	06745	1.9	2.0	–	1.6	1.8	–	–	1.5	–
AA7	GOOX	N	09065	−3.7	−3.8	–	−5.7	−6.5	–	–	3.7	4.4
AA7	GOOX	N	10,788	5.2	5.1	–	3.3	4.2	–	–	1.6	–
AA7	GOOX	N	13,975	–	–	–	−3.0	−1.7	–	–	3.8	3.0
AA9	LPMO	Y	02255	–	3.5	–	--	3.7	–	–	−3.8	–
AA9	LPMO	Y	03338	−1.0	−1.3	–	−1.3	−1.4	–	–	–	–
AA9	LPMO	Y	06603	–	3.4	–	--	5.0	–	–	–	–
AA9	LPMO	Y	08464	–	−1.1	–	–	–	–	–	–	–
AA9	LPMO	Y	10,266	–	5.5	–	6.2	9.3	–	–	−7.0	−4.8
AA9	LPMO	Y	14,047	−1.8	−2.2	–	−1.8	−2.2	–	–	–	–
AA9;CBM1	LPMO	Y	05218	–	6.1	5.8	4.5	7.5	–	–	–	–
AA9;CBM1	LPMO	Y	09248	–	5.7	–	–	6.2	–	–	–	–
AA9;CBM1	LPMO	Y	09249	–	3.9	--	5.8	8.3	–	–	−4.4	--
CBM1;GH74	Xyloglucanase	Y	07273	–	3.4	--	3.9	5.3	–	–	−3.9	–
CBM1;CE1	Acetylxylan esterase	Y	12,496	–	6.6	5.3	3.8	6.2	–	–	–	–
CBM1;CE15	Glucuronoyl methylesterase	Y	11,487	1.5	3.7	2.2	1.8	3.4	–	–	–	–
CBM1;GH10	Endo-1,4-beta-xylanase	Y	02639	2.4	3.9	–	1.8	2.1	–	–	–	–
CBM1;GH10	Endo-1,4-beta-xylanase	Y	03223	–	4.4	–	3.4	4.5	–	–	−3.0	–
CBM1;GH10	Endo-1,4-beta-xylanase	Y	07975	–	4.9	4.0	--	4.1	–	–	–	–
CBM1;GH131	–	Y	03370	–	5.8	5.8	4.7	7.3	–	–	−5.3	–
CBM1;GH3	Beta-glucosidase	Y	07574	–	–	–	–	–	–	–	1.5	–
CBM1;GH3	Beta-glucosidase	Y	10,512	–	–	–	–	–	–	–	2.0	1.4
CBM1;GH3	Beta-glucosidase	Y	10,634	–	4.8	–	–	5.1	–	–	–	–
CBM1;GH5	Endo-beta-1,5-glucanase	Y	04104	–	4.5	–	–	4.2	–	–	–	–
CBM1;GH5	Beta-mannanase	Y	05890	–	--	–	–	3.8	–	–	−3.9	–
CBM1;GH5	Beta-mannanase	Y	11,268	–	4.6	–	5.3	6.5	–	–	−5.0	–
CBM1;GH5_5	Endo-beta-1,5-glucanase	Y	12,259	−3.8	−3.0	–	−1.9	−4.5	−2.5	–	−1.9	–
CBM1;GH6	1,4-beta-cellobiosidase	Y	10,050	–	4.6	3.9	–	3.6	–	–	–	–
CBM1;GH7	1,4-beta-cellobiosidase	Y	04829	–	4.8	–	–	4.6	–	–	–	–
CBM1;GH7	1,4-beta-cellobiosidase	Y	07961	–	--	–	4.7	4.4	–	–	−4.5	−4.0
CBM1;GH7	1,4-beta-cellobiosidase	Y	12,864	–	4.2	–	5.1	6.4	–	–	−4.6	--
CBM20;GH13	Alpha amylase	N	09188	1.4	1.4	–	3.7	4.3	–	–	−1.9	−2.5
CBM20;GH15	Glucoamylase	N	04186	–	3.2	–	3.7	5.8	–	–	−2.5	−2.7
CBM20;GH15	Glucoamylase	N	04190	–	--	--	3.9	2.6	–	−2.1	−5.2	−3.6
CBM20;GH15	Glucoamylase	N	12,151	–	–	–	–	–	–	–	1.2	1.1
CBM60;GH11	Endo-1,5-beta-xylanase	Y	02619	–	–	–	–	–	–	–	−1.9	–
CE1	--	N	00476	2.3	2.5	–	2.0	2.0	–	–	–	–
CE4	Polysaccharide deacetylase	N	01633	−3.4	−3.9	–	−3.9	−4.3	–	1.1	1.7	1.6
CE4	Polysaccharide deacetylase	N	02924	–	–	–	−1.1	−1.1	–	–	--	–
CE4	Polysaccharide deacetylase	N	03495	−2.5	−2.9	–	−3.3	−2.7	–	–	2.2	–
CE4	–	N	06517	4.0	4.8	–	2.9	4.0	–	–	–	–
CE4	Polysaccharide deacetylase	N	11,812	−1.5	−1.5	–	−1.7	−1.5	–	–	–	–
CE8	Pectinesterase	N	04913	1.6	3.0	–	–	–	–	–	–	1.8
GH12	Endo-xyloglucanase	Y	13,789	6.3	7.2	–	6.2	7.3	–	–	–	–
GH12	Endo-xyloglucanase	N	04541	–	4.9	–	3.9	5.8	–	–	–	–
GH12	Endo-xyloglucanase	N	12,231	–	1.4	–	1.5	2.1	–	–	–	–
GH13	Alpha amylase	Y	13,578	−2.0	−1.7	–	−1.1	−1.0	–	–	–	–
GH13	Alpha amylase	N	01055	−1.7	−1.5	–	−1.1	−1.3	–	–	–	–
GH13	Alpha amylase	N	04133	–	–	–	–	–	–	2.4	2.7	2.7
GH13	Alpha amylase	N	12,213	−2.3	−2.7	–	−2.5	−2.8	–	–	–	–
GH13;GH152	Alpha amylase	N	12,203	3.8	3.2	–	2.2	2.4	–	–	–	–
GH13;GT4;GT5	Starch synthase	N	03470	−1.3	−1.3	–	–	–	–	–	–	–
GH13;GT5	Starch synthase	N	04657	1.4	1.7	–	1.4	1.5	–	–	–	–
GH15	Glucoamylase	Y	04370	–	–	–	10.7	9.9	–	–	−7.4	–
GH15	Glucoamylase	N	13,979	1.3	1.3	–	–	–	–	–	–	–
GH27	Alpha-galactosidase	N	05323	--	2.0	2.1	2.1	3.1	–	–	−2.6	−1.5
GH27	Alpha-galactosidase	N	09625	1.4	2.7	–	–	1.4	–	−2.1	−1.9	--
GH28	Polygalacturonase	Y	03020	–	3.4	2.0	1.5	1.6	–	–	–	2.2
GH28	–	Y	05940	–	2.1	–	1.2	1.4	–	–	–	1.3
GH28	Polygalacturonase	Y	06973	2.9	6.4	–	3.2	4.4	–	–	–	–
GH28	Polygalacturonase	Y	08349	–	–	–	–	–	–	2.2	–	2.9
GH28	–	Y	12,986	3.2	4.4	–	1.6	1.5	–	–	–	1.7
GH28	--	Y	13,684	1.9	3.2	–	1.4	1.6	–	–	–	1.1
GH28	Rhamnogalacturonase	N	02734	–	1.6	–	1.9	2.8	–	–	–	–
GH28	–	N	02894	–	3.1	–	–	–	–	–	–	2.2
GH28	Polygalacturonase	N	06016	–	–	–	–	–	–	–	−4.9	–
GH28	–	N	08820	–	–	–	–	3.3	–	–	–	–
GH3	Xylan 1,5-beta-xylosidase	Y	05156	--	4.1	3.3	1.5	2.1	--	--	--	--
GH3	Beta-glucosidase	Y	06168	1.3	1.3	–	1.5	1.4	--	--	--	--
GH3	Beta-glucosidase	Y	06170	1.5	2.5	–	1.3	1.9	--	–	–	–
GH3	Beta-glucosidase	N	11,660	–	1.7	–	–	2.0	–	–	–	–
GH5_5	Endo-beta-1,5-glucanase	N	08136	–	5.5	–	3.1	3.6	–	–	–	–
GH5_50	Glucan 1,3-beta-glucosidase	N	07850	1.9	2.0	–	1.2	1.9	–	–	–	–
GH5_9	Glucan 1,3-beta-glucosidase	Y	11,040	−2.3	−2.8	–	−1.8	−2.2	–	–	–	–
GH9	Endo-beta-1,5-glucanase	N	08227	−1.4	−1.7	–	−1.0	−1.3	–	–	–	–
PL1	Pectate lyase	N	04946	–	–	–	2.8	–	–	–	−3.4	–
PL4	--	Y	10,811	–	–	–	2.3	2.4	–	–	−2.0	−2.0
PL7_4	Alginate lyase	Y	04142	1.9	3.2	1.4	1.3	1.6	–	−1.8	−1.3	–
PL7_4	Alginate lyase	Y	12,410	–	–	–	−1.5	−1.1	–	–	–	–

a SP, Secreted protein.

b The prefix ‘LE01Gene’ of Gene ID has been omitted. LPMO, Lytic polysaccharide monooxygenase.

GOOX, glucooligosaccharide oxidase. ‘--’, no value or no annotation. The grid background are colored according to the value of fold-change.

Most of the LDEs genes were upregulated in sections 2 and 3 on both substrates (Table 1; Supplementary Figures S4–S6; Supplementary Data S4). Two multicopper oxidase genes (AA1, laccase1, LE01Gene08330; laccase11, LE01Gene06108) and one manganese peroxidase (AA2, LE01Gene08997) were simultaneously upregulated on corn stalk and oak wafer. The lignin-degrading auxiliary enzyme (LDAE) genes, related to the breakdown of lignin derivatives, including aryl-alcohol oxidase (AA3_2a, LE01Gene04820, LE01Gene04836), glucose oxidase (AA3_2b, LE01Gene07621), pyranose oxidase (AA3_4, LE01Gene02678) and glyoxal oxidase (AA5_1, LE01Gene02092) showed a strong up-regulation in sections 2 and 3 on both substrates. The expression of genes coding for the core set of enzymes targeting crystalline cellulose (e.g., endoglucanase, GH5-5; cellobiohydrolase (CBH), GH6 (CBH II), LE01Gene10050, GH7 (CBH I), LE01Gene04829, LE01Gene12864, LE01Gene07961), hemicellulose (e.g., GH10-CBM1 and GH11 endo-1,4-beta-xylanase, GH3 β-xylosidase, and CE1-CBM1 acetylxylan esterase) was strikingly upregulated in sections 2 and 3 on both substrates, especially on corn stalk, indicating effective degradation of PCW polysaccharides despite of its high content of soluble sugars. In addition, six genes for copper-dependent lytic polysaccharide monooxygenases (AA9 and AA9-CBM1, LPMO), known to be associated with the depolymerization of cellulose (Ruiz-Dueñas et al., 2021), were significantly up-regulated on sections 2 and 3 of corn stalk; another three LPMO genes (LE01Gene08464, LE01Gene14047, LE01Gene08744) were strongly induced along the whole degradation process on both substrates (Supplementary Data S1). It is worthy to note that a set of (hemi-)cellulase genes, containing the CBM1 domain, and amylase genes, containing the CBM20 domain, were highly induced on corn stalk at a later decay stage (Table 1; Supplementary Data S1). For pectin degradation, similar expression patterns were identified between the two substrates with the exception of several polygalacturonase (GH28, LE01Gene06016, LE01Gene08820, LE01Gene06973, LE01Gene03020) and pectate lyase (PL1 and PL4, LE01Gene04946 and LE01Gene10811) (Supplementary Figure S7). Besides, one alpha-amylase gene (GH13, LE01Gene09188) and three glucoamylase genes (GH15, LE01Gene04186, LE01Gene04190, LE01Gene04370) were significantly up-regulated on corn stalk at the late decay stage (Table 1; Supplementary Figure S7). In general, the higher transcript level of polysaccharide degradation-related genes suggested that corn stalk supplied more carbon sources for hyphal growth.

### Differential expression of genes related to carbohydrate and N metabolism

In addition to the genes directly related to lignocellulose degradation, other genes related to C and N utilization showed significant changes in expression over the decay process. Most genes associated with carboxylic acid transmembrane transport were significantly up-regulated in sections 2 and 3, especially on corn stalk slices, suggesting an important role of carboxylic acid during the substrate degradation (Supplementary Figure S7; Supplementary Data S1). However, higher transcript levels of oxalate decarboxylase and formate dehydrogenase genes were expressed on oak wafers compared with corn stalk slices in sections 2 and 3. Therefore, the degradation of oxalic acid likely reduces the hyphal damage during the colonization of oak wood with tight structure and low accessible nutrient. Considering the strikingly different appearance of hyphae on oak wafers and corn stalk slices, we assessed the transcript level of ribosomal protein genes. A slightly higher transcription level on corn stalk, suggests more active cellular protein synthesis and thus may contribute to the different hyphal morphology and growth rate (Supplementary Data S1). Besides, most genes encoding the oligopeptide transporters (OPT7 and OPT3) and urea transporter (DUR3) in response to N limitation were significantly up-regulated at the late degradation stage for both substrates, particularly on oak wafer (Supplementary Figure S7; Supplementary Data S1). This result suggested that more available N was released from the decomposed corn stalk (Vanden Wymelenberg et al., 2009; Nicolas et al., 2019).

### Enzyme activities related to lignocellulose degradation

**Figure 3:**
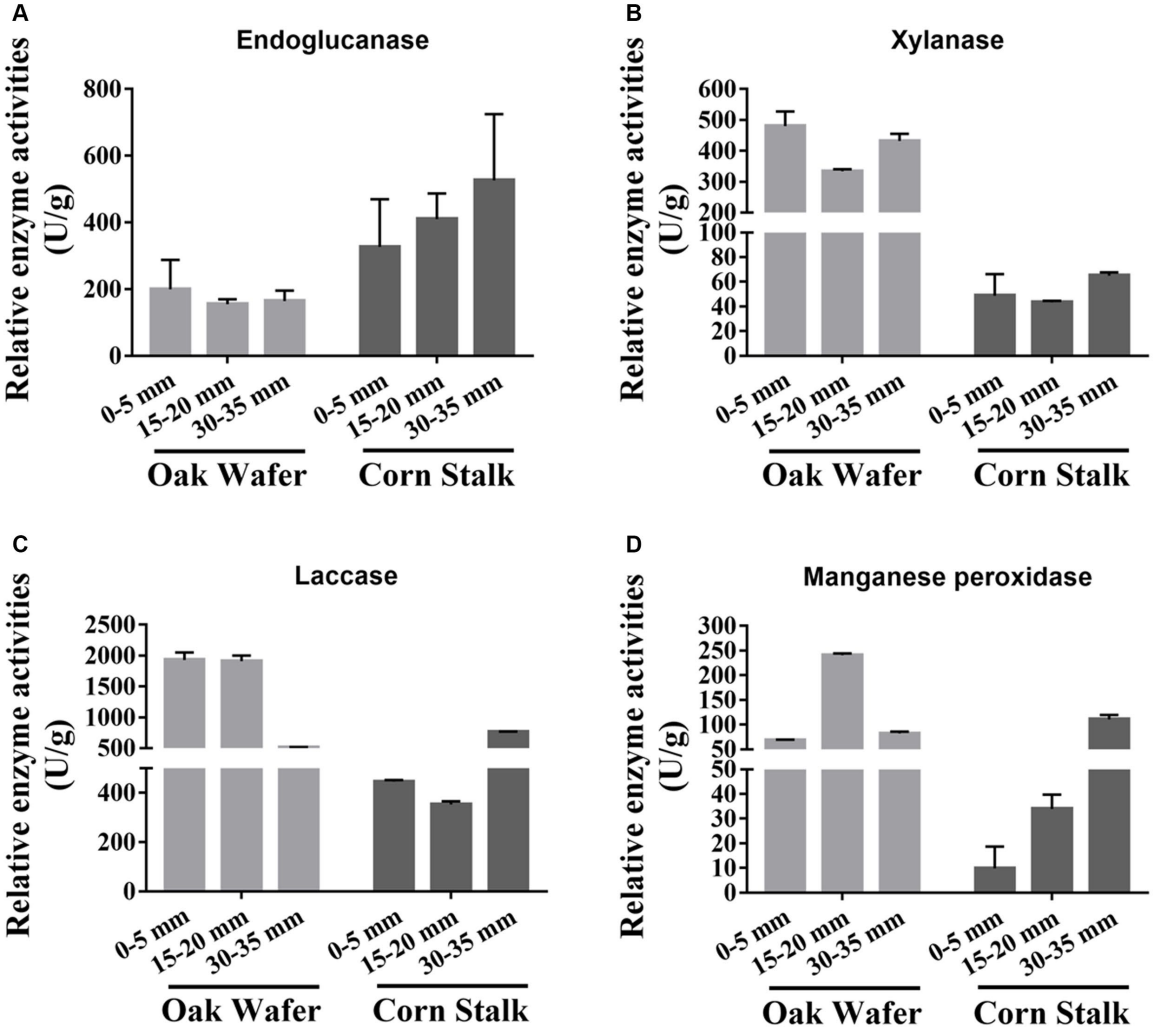
Relative activities of four extracellular enzymes secreted by *L. edodes* hyphae during substrate colonization. The four activities, including endoglucanase **(A)**, xylanase **(B)**, laccase **(C)**, and MnP **(D)**, were measured as units per milligram of total fungal protein and were normalized for each enzyme. Error bars represent the SD of two replicates of those taken from pooled enzyme solutions which contained 15 and 8 hyphae-covered sections of oak wafer and corn stalk, respectively. Section locations for both substrates: 0–5 mm (section 1), ow_1 or cs_1; 15–20 mm (section 2), ow_2 or cs_2; 30–35 mm (section 3), ow_3 or cs_3.

To validate the changes in transcript levels for genes related to lignocellulose degradation, we assayed the activity of a series of extracellular enzymes, that is endoglucanase, xylanase, MnP, and laccase (Figure 3). Briefly, except for the endoglucanase, three out of the four extracellular enzymes showed higher activity on oak wafer than on corn stalk. And the enzyme activities were highly correlated to the gene expression level except for xylanase. The lower activities of endoglucanase on oak wafer might be attributed to the lower expression of endoglucanase genes (Table 1; Supplementary Figure S5). In contrast, the higher activity of laccase might contributed by several genes, such as lcc9 (LE01Gene04648), lcc12 (LE01Gene08149) and lcc4 (LE01Gene04008) (Table 1; Supplementary Figure S4). MnPs activity might contributed by gene LE01Gene09060, the only exocellular enzyme of MnPs (Table 1). Besides, the activity of xylanase was not in line with the gene expression level, which was similar to previous findings, and the reason might be complicated (Daly et al., 2018).

### Changes in the substrate surface structure

**Figure 4:**
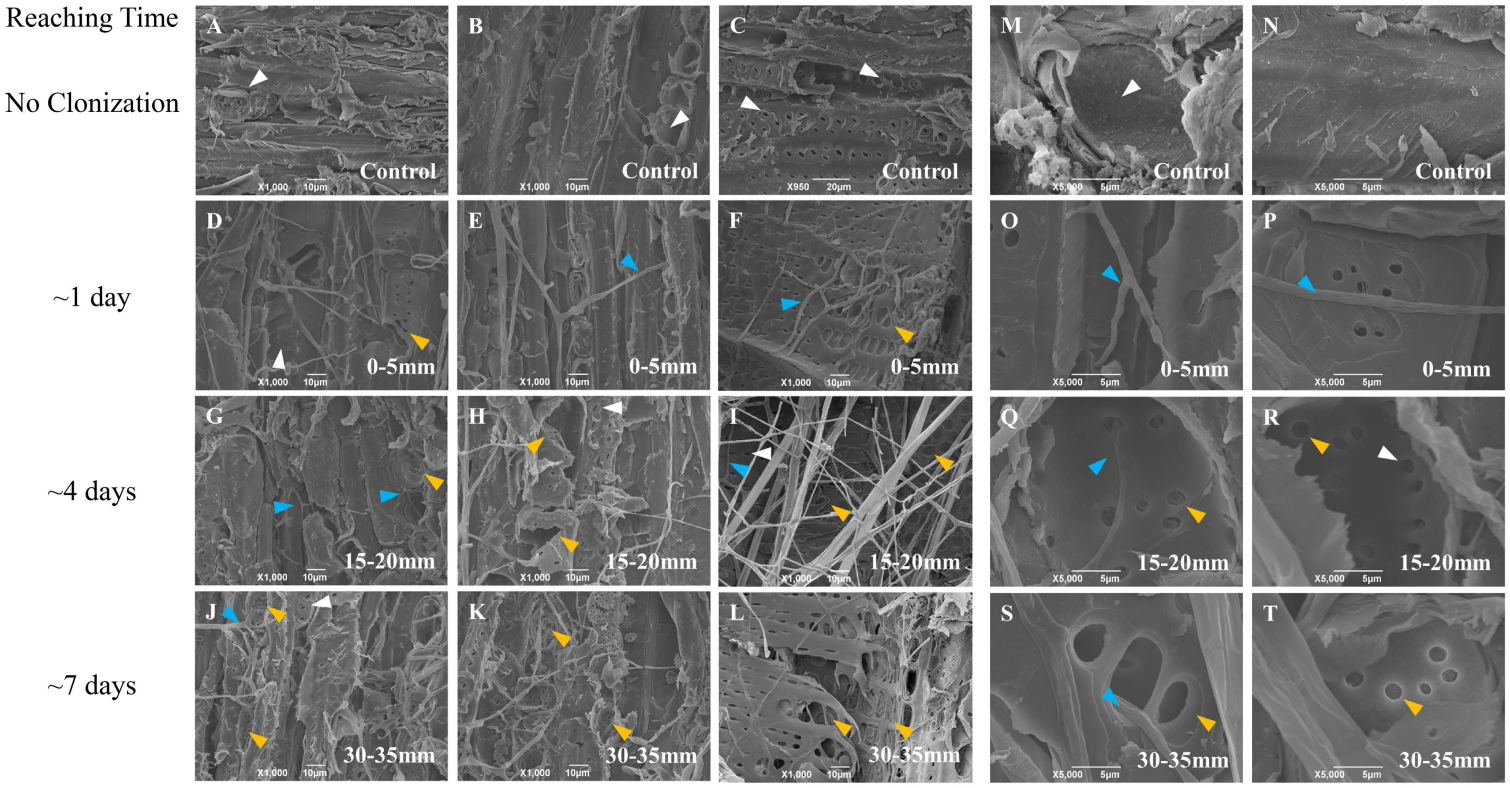
SEM observation of the surface of vertical oak wafer decayed by *L. edodes* hyphae. **A–C** and **M–N**, non-inoculated substrate sections; **D–F** and **O–P**, substrate sections at 0–5 mm (distance from the hyphal-colonization front); **G–I** and **Q–R**, substrate sections at 15–20 mm; **J–L** and **S–T**, substrate sections at 15–20 mm. The white arrow indicates the original pits and structure, the orange-yellow arrow indicates the decayed or dissolved pits and structure, and the blue arrow indicates the hyphae. Section locations for both substrates: 0–5 mm (section 1), ow_1 or cs_1; 15–20 mm (section 2), ow_2 or cs_2; 30–35 mm (section 3), ow_3 or cs_3. The reaching time of hyphal colonization has been added on the right side of the figure.

**Figure 5:**
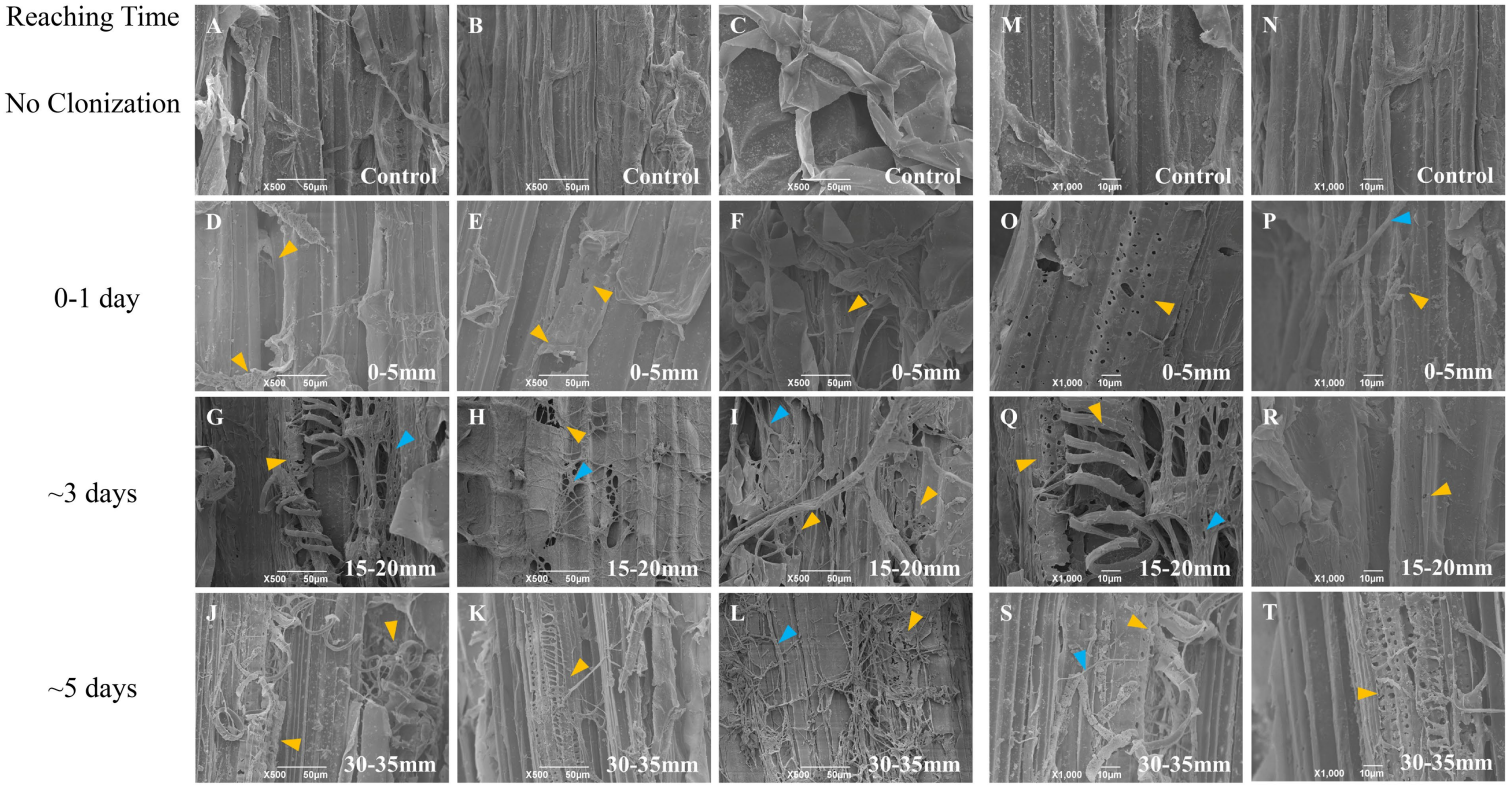
SEM observation of the surface of vertical corn stalk slice decayed by *L. edodes* hyphae. **A–C** and **M,N**, non-inoculated substrate sections; **D–F** and **O,P**, substrate sections at 0–5 mm (distance from the hyphal-colonization front); **G–I** and **Q,R**, substrate sections at 15–20 mm; **J–L** and **S,T**, substrate sections at 15–20 mm. The orange-yellow arrow indicated the decayed structure and the blue arrow indicated the hyphae. Section locations for both substrates: 0–5 mm (section 1), ow_1 or cs_1; 15–20 mm (section 2), ow_2 or cs_2; 30–35 mm (section 3), ow_3 or cs_3. The reaching time of hyphal colonization has been added on the right side of the figure.

Considering the different structures and anatomy of oak wood wafer and corn stalk slice, we characterized their vertical surface at three locations (0–5 mm, 15–20 mm, 30–35 mm) with SEM (Figures 4, 5). The intact structure could be seen on the surface of untreated oak wafer and corn stalk, which exhibited different morphologies. The longitudinally surface of oak wafer was mainly composed of large vessels of tracheids with some pits and perforations in the cell wall, smaller cavities of libriform cells, and some other ray cells. By contrast, corn stalk consisted of cortexes and a large amount of piths composed of square parenchyma cells and scattered vascular bundles. The square parenchyma cells were connected closely to each other with a fluffy structure.

Interestingly, the hyphae attached and colonized preferentially to the inside wall of the lumen (secondary cell wall) of oak wafers and perforated the pits on the internal surface of the cell wall during the spatial–temporal degradation process (Figure 4). Following the degradation by hyphae, the surface of both substrates became increasingly rough, particularly the oak wafer surface. Then, substrate debris adhered to each other likely through the action of secreted enzymes. For corn stalk, the surface showed increases in pores and debris, the xylem tissue was destroyed and some helical structure was released after degradation (Figure 5). And finally, the corn stalk became soft and spongy. Besides, some oxalate crystal was deposited on the oak wafer surface (Supplementary Figure S8) and likely involved in metal chelation, pH regulation, and even metal detoxication (Gadd, 1999). In general, hyphae could efficiently degrade both corn stalk and oak wafer but with different degradation morphological features.

### Substrate compositional change during hyphal colonization

**Figure 6:**
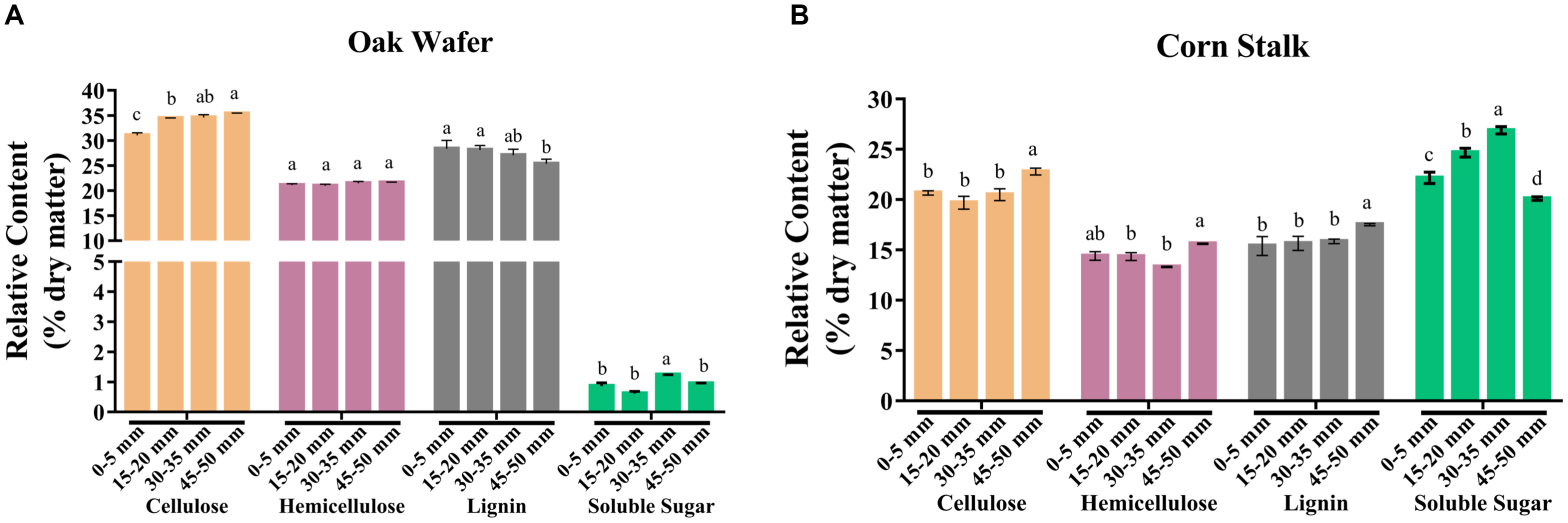
Relative content of four major compounds (cellulose, hemicellulose, lignin, and soluble sugar) of substrate sections of oak wafer **(A)** and corn stalk **(B)** during degradation. Error bars represent the SD of three replicates. The significance of the difference in relative content has been labeled by lowercase letters along the various stages of *L. edodes* colonized substrate by t-test at *p* < 0.05 (*n* = 3). Section locations for both substrates: 0–5 mm (section 1), ow_1 or cs_1; 15–20 mm (section 2), ow_2 or cs_2; 30–35 mm (section 3), ow_3 or cs_3.

To better understand the cell wall degradation of oak wafer and corn stalk, we measured the content of four major components (cellulose, hemicellulose, lignin, and soluble sugar) and three lignin monomers in the three substrate sections. Considering the short time of colonization, the composition of the substrate in section 1 represented the initial substrate composition. Based on the dry matter, the relative content of cellulose, hemicellulose, lignin and soluble sugar in section 1 was 31.08, 21.10, 28.39 and 0.86% in oak wafer (Figure 6A). It was 20.68, 14.41, 15.40, 22.16% in corn stalk (Figure 6B). In particular, corn stalk had 22% higher soluble sugar than oak wafer. These results are similar to previous findings for sweet sorghum, corn stalk, and oak wood (Jia et al., 2014; Li et al., 2014a; van Kuijk et al., 2017).

After colonization and degradation by *L. edodes*, the four major components exhibited different degradation in the different spatial–temporal sections of oak wafers and corn stalk slices. The proportion of lignin in oak wafer significantly decreased, while cellulose was increased. This result is highly consistent with the high transcript level and enzyme activity of lignin degradation related genes on oak wafer. In contrast, a slight decrease of cellulose and hemicellulose in corn stalk was detected from section 1 to section 3. In section 4, the significant decrease in soluble sugar may contribute to the increase of cellulose, hemicellulose, and lignin. These results showed that lignin in oak wafer and cellulose and hemicellulose in corn stalk were mainly degraded, but not by much.

### The change of three lignin monomers

**Figure 7:**
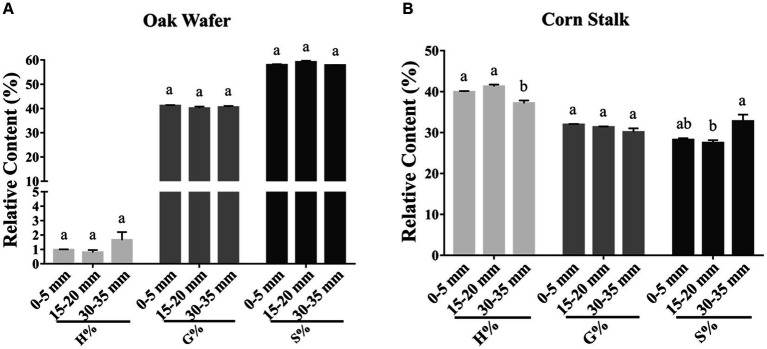
Relative content of three lignin monomers (H, G, and S) in substrate sections of oak wafer **(A)** and corn stalk **(B)** during degradation. Error bars represent the SD of two replicates. The significance of the difference in relative content has been labeled by lowercase letters along the various stages of *L. edodes* colonized substrate by *t*-test at *p* < 0.05 (*n* = 2). Section locations for both substrates: 0–5 mm (section 1), ow_1 or cs_1; 15–20 mm (section 2), ow_2 or cs_2; 30–35 mm (section 3), ow_3 or cs_3.

To assess whether the three lignin monomers were degraded at the same rate, we estimated their relative proportions by HPLC. As shown in Figure 7, oak wafer lignin monomers mainly include G- (41.12%) and S- (57.91%) and trace amounts of H- monomer (0.97%). By contrast, the corn stalk has the highest level of H- (39.90%), followed by G- (31.90%) and S- monomer (28.20%).

The proportions of the three lignin monomers in oak wafer showed no significant change during the time-spatial degradation process (*t*-test). Despite no obvious reduction in the lignin proportion of corn stalk, the proportion of G-monomer showed a slight but continuous (not significant) decrease in corn stalk. In addition, there were sequential changes in S- and G-monomer in section 2, followed by significant degradation of H-monomer, suggesting the sequential degradation of the three monomers in corn stalk and that the lignin structure in corn stalk is different from that in oak wafer.

## Discussion

In this study, the spatial–temporal colonization showed a higher extension rate and thicker hyphae appearance on corn stalk, possibly because of its much higher content of accessible nutrients, such as soluble sugars, for the initial substrate colonization (van Kuijk et al., 2017). Correspondingly, different degradation strategies were employed during the spatial–temporal colonization on these two solid substrates. In the initial colonization within 24 h, higher activities of oxidases were observed on oak wafer compared with on corn stalk, and the different transcriptional levels related to ions and organic acids transportation were also identified, these results suggest that the strategy of substrate degradation has been launched at a very early stage. After the initial response to the substrate and removal of the small molecule compound, the rest part of the substrate, including the high polymer constituting the main component of the cell wall, need a series of extracellular enzyme to continuously depolymerize and then further metabolize in the cell (Chen et al., 2016). On both substrates, a significant shift of gene expression between the hyphal-colonization front and backend was presented as similar to the shift of gene expression in brown-rot fungi (Zhang et al., 2016). However, a progressive expression pattern involved in substrate oxidation and hydrolysis was observed in *L*. *edodes*, and different from the staggered gene expression pattern in brown-rot fungi, which may be attributed to the different extension rate of hyphae along the substrate wafer and rot type (Zhang et al., 2016; Presley et al., 2018). And this spatial–temporal colonization with different substrates in solid condition better reflects the depolymerization of the PCW. For example, the higher gene expression and enzyme activities of MnP and laccase in line with the preferential degradation of lignin on oak wafer at the initial stage, suggest a preferential degradation of lignin (Cai et al., 2017; van Kuijk et al., 2017). However, this priority was been delayed on corn stalk because of the different substrate compositions. Besides, the simultaneously and highly induced enzymes might form a sophisticated enzyme system under a given substrate environment (Zhu et al., 2016; Zhang et al., 2019). In general, a sophisticated and flexible expression and secretion system of lignocellulose degrading enzyme was employed according to the complexity of composition and accessibility of nutrition during the colonization of oak wafer and corn stalk for *L. edodes* mycelium.

The up-regulated genes of laccase in section 1 suggest that lignin degradation occurred at the hyphal-colonization front (Srebotnik and Messner, 1994; van Kuijk et al., 2017). Laccases, with a low redox potential, are often expressed at an early stage of colonization and mainly attack the phenolic lignin, a minor part of lignin (Martínez et al., 2005). However, the continuously high level of several genes of laccases and MnPs in sections 2 and 3 indicated a sustained process of lignin degradation (Zhang et al., 2019) or detoxification occurring during the whole degradation process (Morel et al., 2013). The co-expressed enzymes of (hemi-)cellulases, lignin oxidases, and LDAEs in sections 2 and 3, indicate the concurrent depolymerization of cell wall components (Zhu et al., 2016). In addition, the similar expression patterns of hemicellulase and pectinase genes supported that hemicellulose and pectin are simultaneously degraded to expose and degrade cellulose (Pérez García et al., 2011; Presley et al., 2018).

Besides, our spatial–temporal transcriptomic profiling also revealed different responses and expression patterns, including enzyme genes of oxidation and hydrolysis, due to the different substrate types and even compositions (Daly et al., 2018). A previous study reported that MnPs are involved in the lignin degradation of solid wood substrate (Daly et al., 2018). A higher content of Mn^3+^ chelates may be required to penetrate the tight wood structure of oak wafers (containing denser G-monomer) compared to grass type structure, such as corn stalk (Mac Donald et al., 2011; van Kuijk et al., 2017). The CBM1 domain is often required to bind the enzyme to the glucan chain (Watkinson, 2016), thereby preventing the enzyme from leaching in a loose environment such as leaf litter compared with compact wood (Ruiz-Dueñas et al., 2021). For example, the grassland-inhabiting *P. eryngii* encodes more CBM1-containing enzymes than the wood-rotter *P. ostreatus* in Agaricales (Ruiz-Dueñas et al., 2021). Corn stalk composition and structure are closer to grass litter with soft and spongy structure, and have a higher water-holding capacity. In *Laccaria bicolor*, a symbiotrophic fungi, the presence of the CBM1 domain increases the endoglucanase enzyme activity (beta-1,4-endoglucanase) and thermostability to bind cellulose (Zhang et al., 2018). Consequently, the CBM1 domain can help to bind to the substrate surface and effectively degrade the polysaccharide, especially on corn stalk, at a later decay stage.

The hyphal colonization and substrate degradation were observed at a higher resolution compared with previous studies (Martínez et al., 2005; Zhu et al., 2016; Daly et al., 2018). The decayed substrate surface showed typical features of white rot, including enlarged and perforated pits in the S3 layer of the secondary cell wall (vessel cells and libriform cells) (Ji et al., 2012). Besides, the dissolved-perforation and torn-degradation of vertically cut surfaces at different locations could explain the thinning of secondary cell walls (Ji et al., 2012; van Kuijk et al., 2017; Daly et al., 2018). On oak wafers, oxalic acid was secreted to maintain the activity and stability of lignin-degrading enzymes with a low pH in section 1 (Ayuso-Fernandez et al., 2017; Ayuso-Fernández et al., 2018). Subsequently, most oxalic acid was decarboxylated by oxalate decarboxylase to CO_2_ and formate, and then formate may be dehydrogenated by formate dehydrogenase in the follower section (Shimada et al., 1997). Therefore, it reduced the hyphal damage in the process of colonization and perforation. These results coincide with the highly expressed transcript level of oxalate degradation-related genes in section 3 of the oak wafer (Supplementary Figure S7; Supplementary Data S1).

Compared with that of other components (cellulose and hemicellulose), the proportion of lignin in oak wafer significantly decreased, suggesting preferential degradation, which was also observed by using a combination of Safranin O and Astra Blue staining (Srebotnik and Messner, 1994; Korripally et al., 2015; Fernández-Fueyo et al., 2016). Besides, cellulose was degraded, especially in oak wafer, and perhaps the slight reduction of cellulose in absolute content was masked by the significant increase of its proportion within a short colonization time (van Kuijk et al., 2017; Daly et al., 2018; Ding et al., 2019; de Figueiredo et al., 2021). The significant reduction of soluble sugar in the backend section of corn stalk might be partially attributed to the consumption and transport to the lower hyphae. In general, the substrate components showed a relatively minor decrease compared with those in previous studies, which may be mainly attributed to the short colonization time used in our study (Daly et al., 2018). In addition, oak wafer and corn stalk showed different degradation for three lignin monomers due to the different structure and composition. Taking the significant reduction of lignin proportion together, these results suggested that three lignin monomers were degraded simultaneously in oak wafer. In contrast, the continuous decrease in G-monomer in corn stalk (gramineous plants) likely facilitates the access of enzymes to other cell wall components. According to previous studies, G-monomer in Miscanthus belonging to gramineous plants may play a central role in linking other cell wall components and this view may explain the continuous reduction of G-monomer in corn stalk (Li et al., 2014b).

Despite the much higher soluble sugar content in corn stalk and its continuous increase in the first three sections, higher endoglucanase transcript levels and activities were observed in the hyphae on corn stalk than those on oak wafer, suggesting that the mycelium needs other nutrient components for growth during the colonization process, such as entrapped N compounds (Nicolas et al., 2019). Since no extra nutrient was added in the pure culture system to make it closer to the natural environment, the degradation of the substrate should be not only for obtaining polysaccharides but also for other components. In addition, the wood had an extremely lower N content (C:N ratio up to 1,250:1) (Watkinson and Eastwood, 2012; Hess et al., 2021) than corn stalk (C:N ratio about 50–55:1) (Zhang et al., 2015). Therefore, the up-regulated or sustained high transcription level of genes involved in organic N assimilation, including extracellular peptidases and N transporters, which may be caused by the shortage of N in oak wafer (Nicolas et al., 2019). Besides, the slightly down-regulated genes related to ribosomal protein synthesis on oak wafer optimized the N utilization by reducing the ribosomal gene expression compared with in corn stalk in sections 2 and 3 (Hess et al., 2021). For these genes with high transcript levels, some subtle changes in the use of codons will affect the N investment of hyphal cells (Kelly, 2018). With the simultaneous degradation of lignin and (hemi-)cellulose and sustained lignin degradation, white-rot fungi require stricter regulation of gene expression to save energy (Zhang et al., 2019). Interestingly, only a few genes in the multigene families related to the degradation of lignocellulose maintained high expression, especially on oak wafer, which may be a trade-off strategy to reduce energy investment under nutrient-limited conditions (Zhang et al., 2019; Floudas et al., 2020). By contrast, many gene paralogs were lost during the evolution of brown rots (Kohler et al., 2015). The remaining paralogous genes generally have high expression and are more tolerant to oxidative radicals (Zhang et al., 2019). Therefore, to better adapt to the N-poor oak wafer, the *L. edodes* hyphae need to reduce the investment of cellular N and thereby present thinner mycelia than on corn stalk.

## Conclusion

Our transcript profiling showed that colonizing *L. edodes* hyphae alter their gene expression patterns in response to different substrate compositions. They mobilized several oxidoreduction pathways during the spatial–temporal colonization on oak wafer with higher content of lignin and related compounds. By contrast, CBM1-containing (hemi-)cellulases played a critical role in the polysaccharide decomposition of corn stalk characterized by their spongy and microporous structure (i.e., high capacity of water storage and a loose substrate environment). In addition, the higher soluble sugar content of corn stalk can serve as an extra carbon source for early fungal colonization and growth. The substrate composition, such as lignin monomers and the content in N compounds, likely modulates the decomposition of lignocellulose. Hence, the spatial–temporal gene expression profile for *L. edodes* and different degradation morphology for oak wafer and corn stalk slice may provide us important guidance about the PCW degradation character of *L. edodes* in the early colonization phase of wood and straw for the effective bio-conversion and utilization of biomass by white-rot fungi.

## Data availability statement

The datasets presented in this study can be found in online repositories. The names of the repository/repositories and accession number(s) can be found below: GSA - CRA010424.

## Author contributions

CM: Conceptualization, Data curation, Formal analysis, Investigation, Methodology, Validation, Visualization, Writing – original draft, Writing – review & editing. YG: Conceptualization, Formal analysis, Funding acquisition, Methodology, Writing – review & editing. HK: Data curation, Formal analysis, Funding acquisition, Project administration, Supervision, Writing – review & editing. LC: Data curation, Investigation, Software, Writing – review & editing. FM: Writing – review & editing. YB: Conceptualization, Data curation, Formal analysis, Methodology, Project administration, Supervision, Writing – review & editing.
