# Supplementary material for: Comparative analysis of simulated in-situ colonization and degradation by Lentinula edodes on oak wafer and corn stalk
Source: Front Microbiol. 2023 Nov 23;14:1286064. doi: 10.3389/fmicb.2023.1286064 (PMC10701760; doi:10.3389/fmicb.2023.1286064)
Supplement: Supplementary file 5 [file Data_Sheet_5.docx]

**Supplementary materials**

**Content index:**

1. **Supplementary methods**
2. **Supplementary data**
3. **Supplementary figures**

**1. Supplementary methods**

**1.1 Preparations for vertically hyphal growth.** The mycelium of commercial dikaryotic strain W1 (ACCC 50926) of L. edodes (Wuhan, China) was maintained on solid MYG medium (20 g/l sucrose, 20 g/l malt extract, 1 g/l yeast extract, 1 g/l peptone, and 15 g/l agar) for 7 d at 25 ℃ in the dark before inoculation of the solid substrates, oak wafer and corn stalk slice. The two substrates were collected from the experimental field and Shizishan (Lion Mountain) of Huazhong Agricultural University, respectively. The corn stalk was cut into ~2 mm slices. Briefly, vermiculite hydrated to 45%–55% (v/v) moisture content was packed into glass bottle with a volume of 375 ml and a depth of ~15 mm and sterilized by autoclaving together with two substrates. Then, oak wafer and corn stalk slice (~60 × ~25 × ~2.5 mm) placed on the vermiculite surface were inoculated with 2-mm diameter agar plugs of L. edodes cultivated on solid MYG medium. When the oak wafer and corn stalk slices were covered by mycelia after 5–7 d, a new oak wafer or corn stalk slice (~70 × ~25 × ~2.5 mm) was vertically placed on the first slices. Then, the agar plugs were removed. After the L. edodes hyphae visibly colonized the substrate in an oriented direction and extended up to ∼50 mm at 25℃, the oak wafer and corn stalk slice were taken out and those with a smooth hyphal front were used to harvest the hyphae.

**1.2 Data processing of transcriptome profiles.** The raw reads were trimmed and filtered using the Trimmomatic software to remove adapter-containing and low-quality reads (Bolger *et al.*, 2014). Clean reads were mapped to the L. edodes strain W1 genome using HISAT2 and Bowtie2 (Kim *et al.*, 2015; Langdon, 2015). The gene expression levels were calculated using the Stringtie software based on the DESeq2 method, and differential expression analysis was performed using DESeq2 (Love *et al.*, 2014). Gene ontology (GO) enrichment of the DEGs was performed using Blast2Go and Phyper based on the Hypergeometric test (Tarazona *et al.*, 2011), respectively. Venn diagrams were generated by VENNY 2.1 (Oliveros, J.C. (2007–2015). The sample similarity, heat maps, and related graphs were generated by SPSS software (SPSS 17.0), Tbtools software (Chen *et al.*, 2020), and GraphPad software (Prism 7.0.0).

**1.3 Enzyme activity determination.** Extracts were centrifuged at 8000 rpm for 5min and the supernatant was used for the enzyme assay. Endoglucanase (CMCase) and xylanase were measured with dinitro salicylic acid reagent, and 0.5% carboxymethyl cellulose and 0.5% birchwood xylan were used as substrates, respectively. The MnP activity was measured by monitoring the change of OD465 in 0.05 M citrate buffer containing 2 mM MnSO4, 0.1 mM H2O2, and 5 × 10-5 mM guaiacol. Laccase activity was measured by monitoring the change of OD420 in 0.05 M citrate buffer with 0.5 mM ABTS as the substrate. The reagents used above for enzyme activity analysis were purchased from Sinopharm Chemical Reagent Co., Ltd. One unit of endoglucanase (CMCase), xylanase, MnP, and laccase activities was defined as the enzyme amount needed for the release of 1 μmol glucose, 1 μmol xylose, and the changes of OD465 and OD420 per min in reaction, respectively, under the same conditions.

**2. Supplementary Data:**

Supplementary file S1. Normalized count of gene expression

Supplementary file S2. Total DEGs identified in *L. edodes* transcriptome by paired comparisons

Supplementary file S3. GO enrichment result of total DEGs

Supplementary file S4. Differential expression of CAZyme genes in *L. edodes* transcriptome during substrate colonization

1. **Supplementary Figures: see below.**


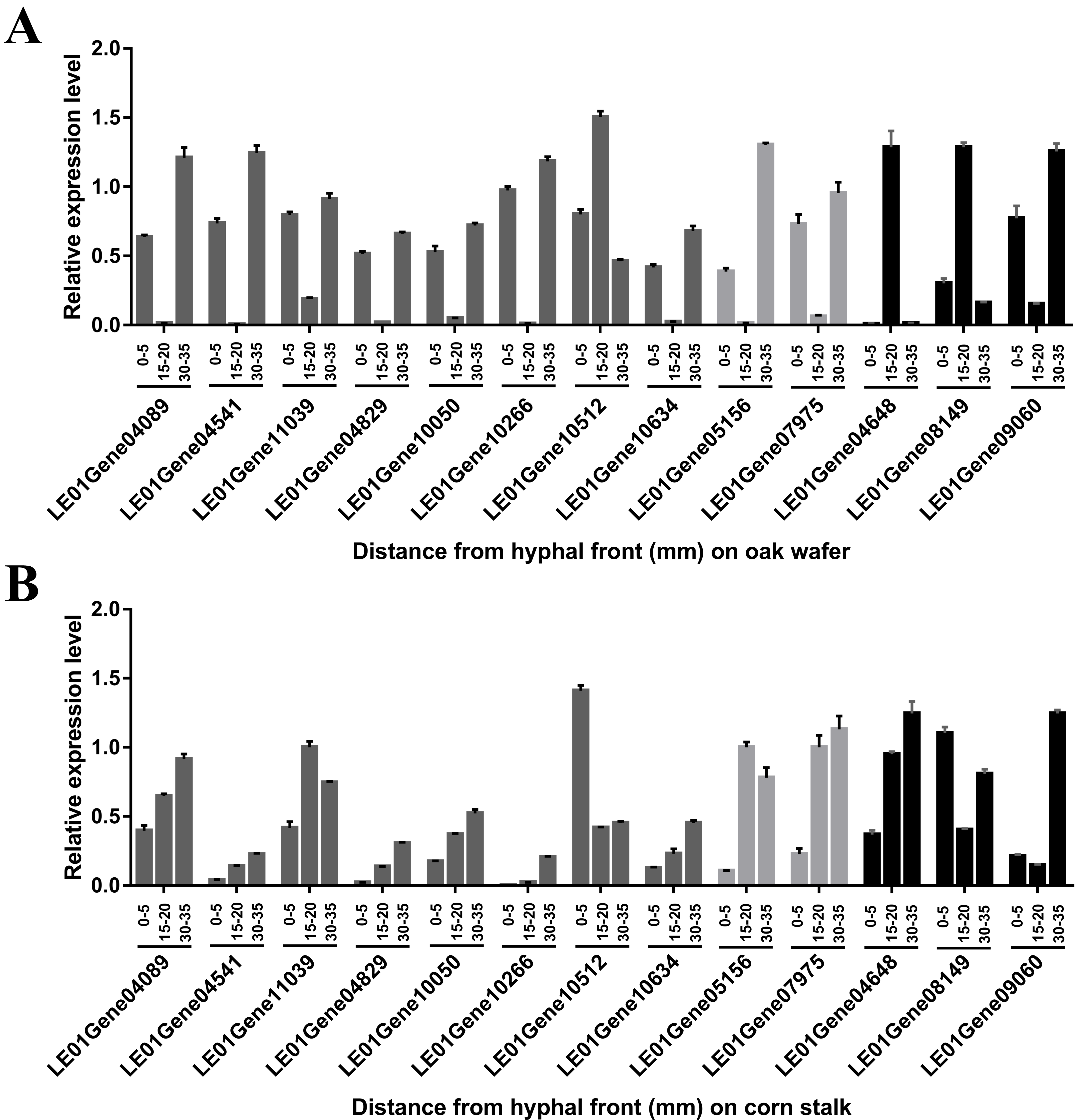


**Fig. S1. Preliminary qPCR analysis of gene expression during substrate colonization.** Gene expression of several lignocellulose degradation-related genes on oak wafer (A) and corn stalk (B). The relative expression levels were normalized with the β-actin gene and the mean values were calculated with three independent biological replicates. The three colors (dark grey, grey, and black) of the bar chart indicate the gene expression of cellulose, hemicellulose, and lignin degradation-related enzymes, respectively. Section locations for both substrates: 0–5 mm (section 1), ow_1 or cs_1; 15–20 mm (section 2), ow_2 or cs_2; 30–35 mm (section 3), ow_3 or cs_3.


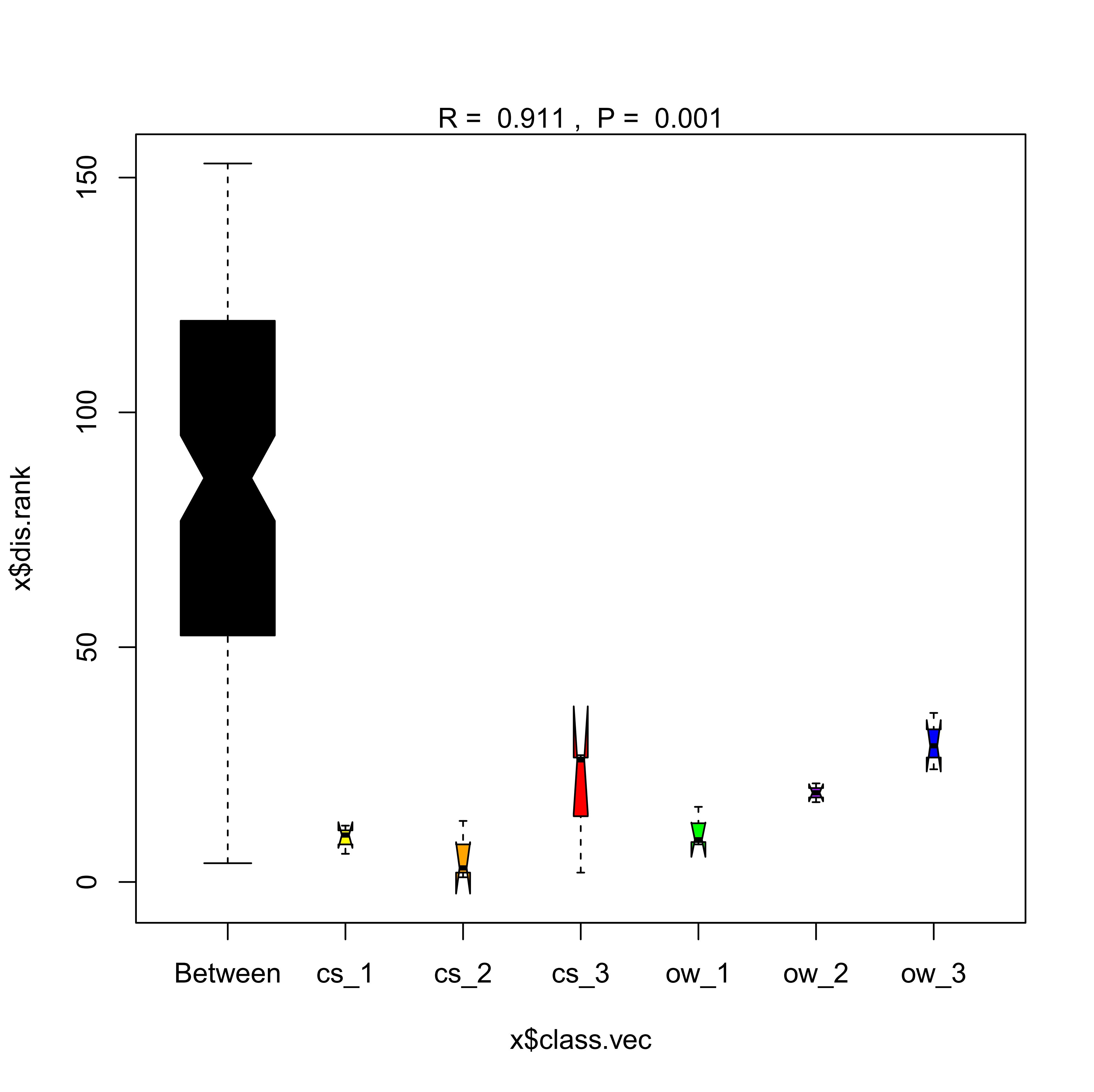


**Fig. S2. ANOSIM of sample groups.**


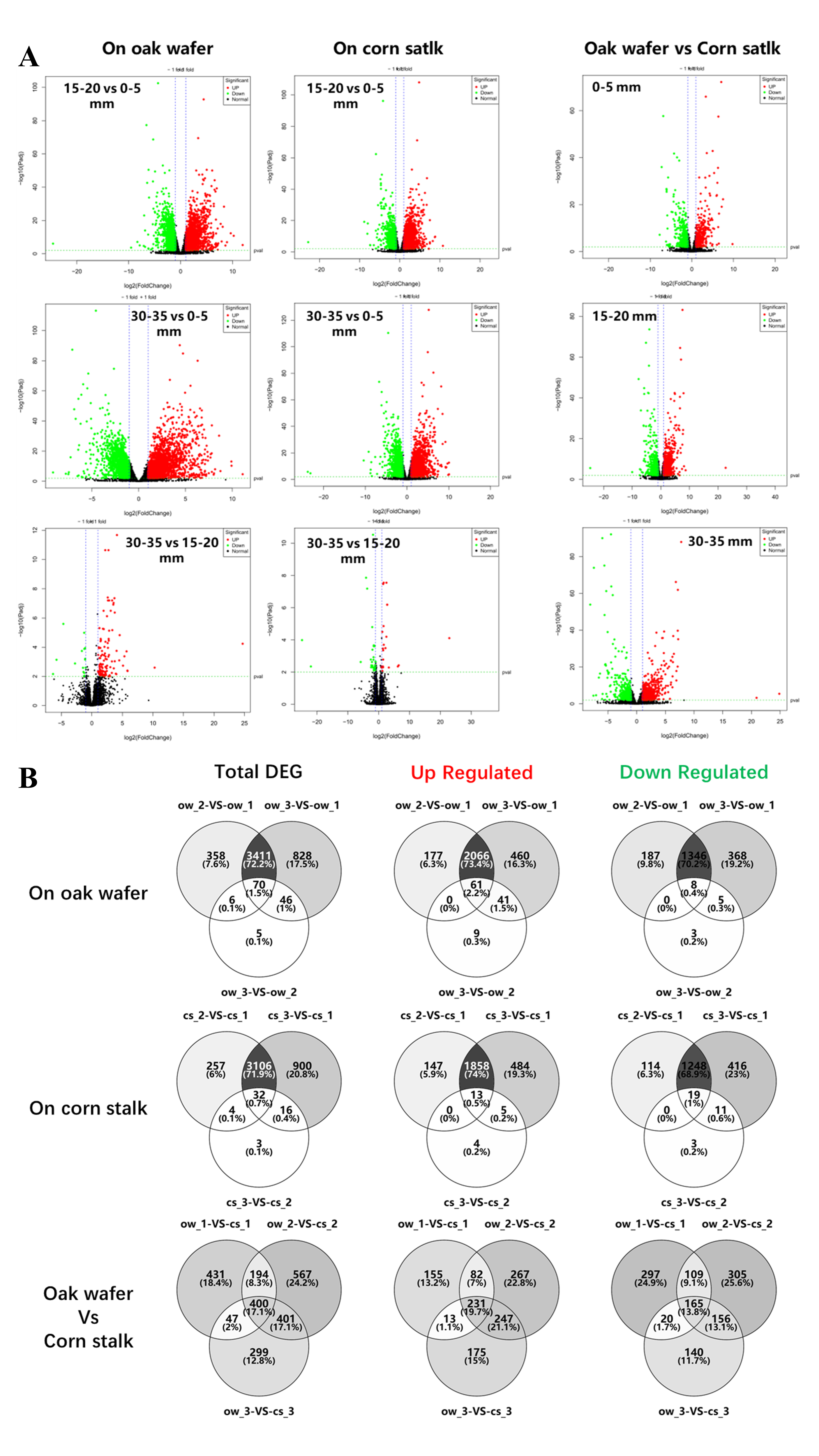


**Fig. S3. Total DEG numbers during substrate colonization.** (A) Volcano plot of paired comparison in *L. edodes* transcriptome and (B) Venn diagram of the spatial-temporal DEGs on different substrates. Section locations for both substrates: 0–5 mm (section 1), ow_1 or cs_1; 15–20 mm (section 2), ow_2 or cs_2; 30–35 mm (section 3), ow_3 or cs_3.


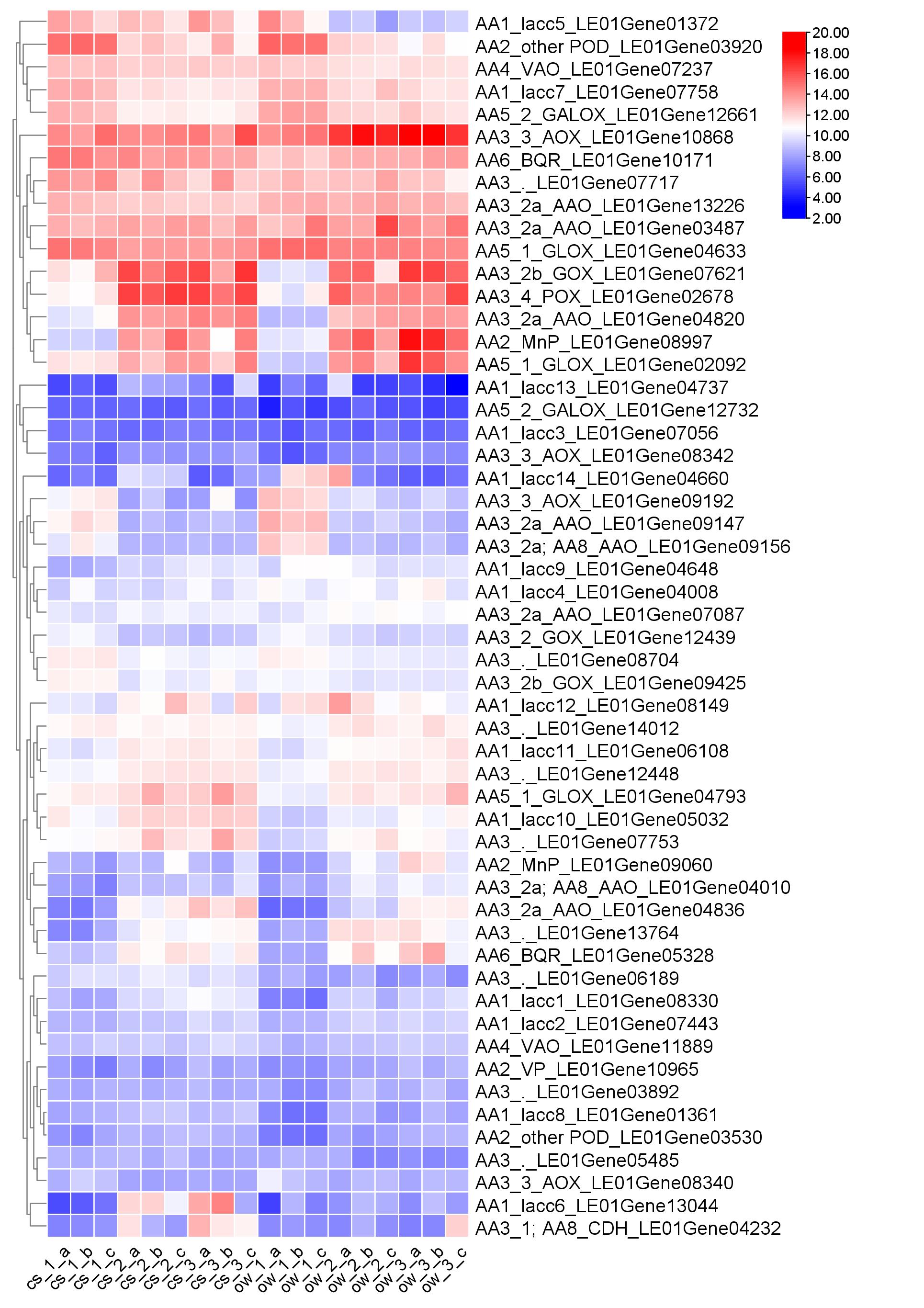


**Fig. S4.** **Expression heatmap of enzyme genes related to lignin oxidation and degradation during the spatial-temporal colonization using log2 (Normalized Count).** Section locations for both substrates: 0–5 mm (section 1), ow_1 or cs_1; 15–20 mm (section 2), ow_2 or cs_2; 30–35 mm (section 3), ow_3 or cs_3. Abbreviation: lacc, laccase; MnP, Manganese peroxidase; other POD, Other peroxidase; VP, Versatile peroxidase; CDH, Cellobiose dehydrogenase; GOX, Glucose oxidase; AAO, Aryl-alcohol oxidase; AOX, Alcohol oxidase; POX, Pyranose oxidase; GLOX, Glyoxal oxidase; VAO, Vanillyl-alcohol oxidase; BQR, Benzoquinone reductase; GALOX, Galactose oxidase.


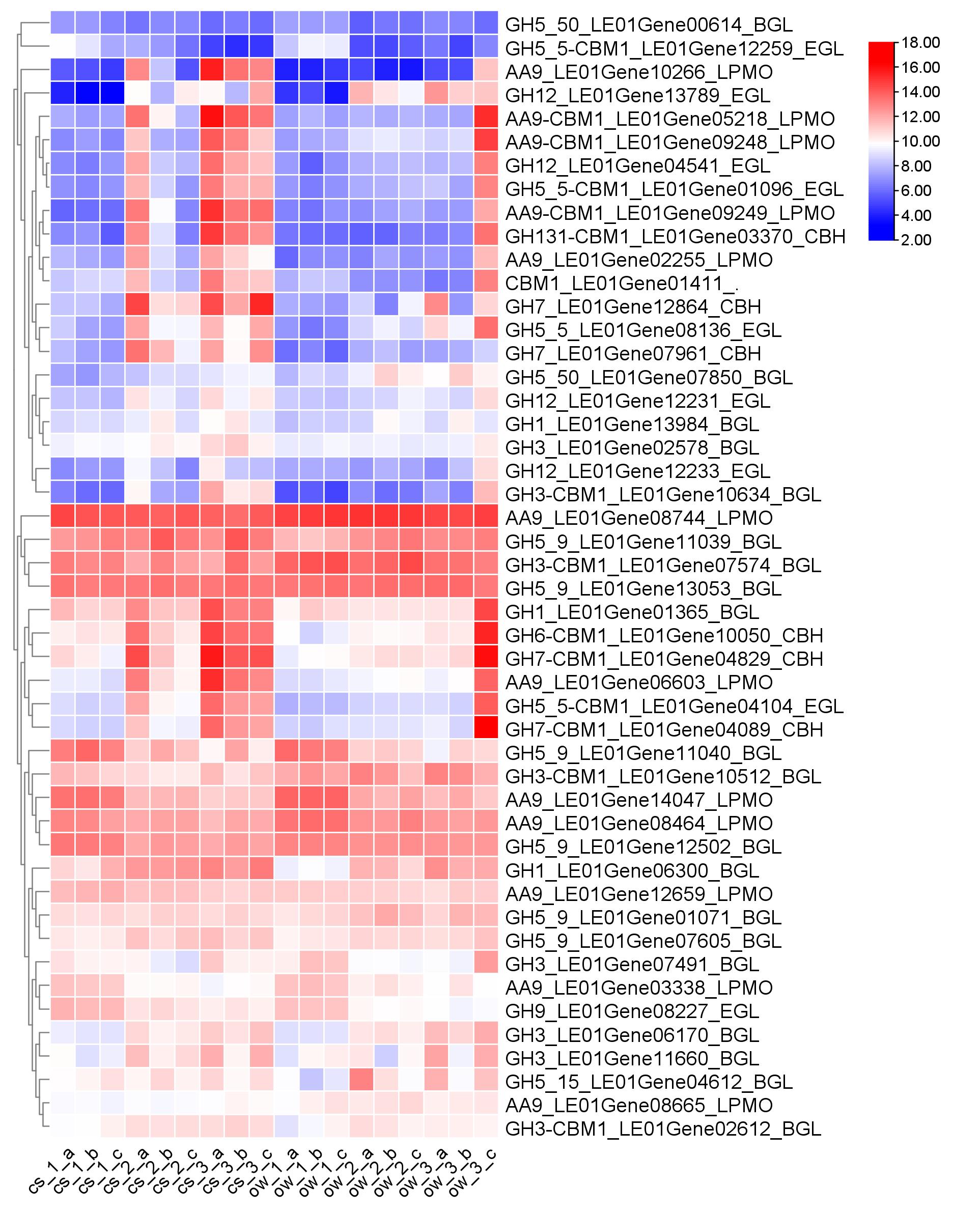


**Fig. S5. Expression heatmap of enzyme genes related to cellulose degradation during the spatial-temporal colonization using log2 (Normalized Count).** Section locations for both substrates: 0–5 mm (section 1), ow_1 or cs_1; 15–20 mm (section 2), ow_2 or cs_2; 30–35 mm (section 3), ow_3 or cs_3. Abbreviation: LPMO, Lytic polysaccharide monooxygenase; BGL, Beta-glucosidase; EGL, Endo-beta-1,4-glucanase; CBH, 1,4-beta-cellobiosidase.


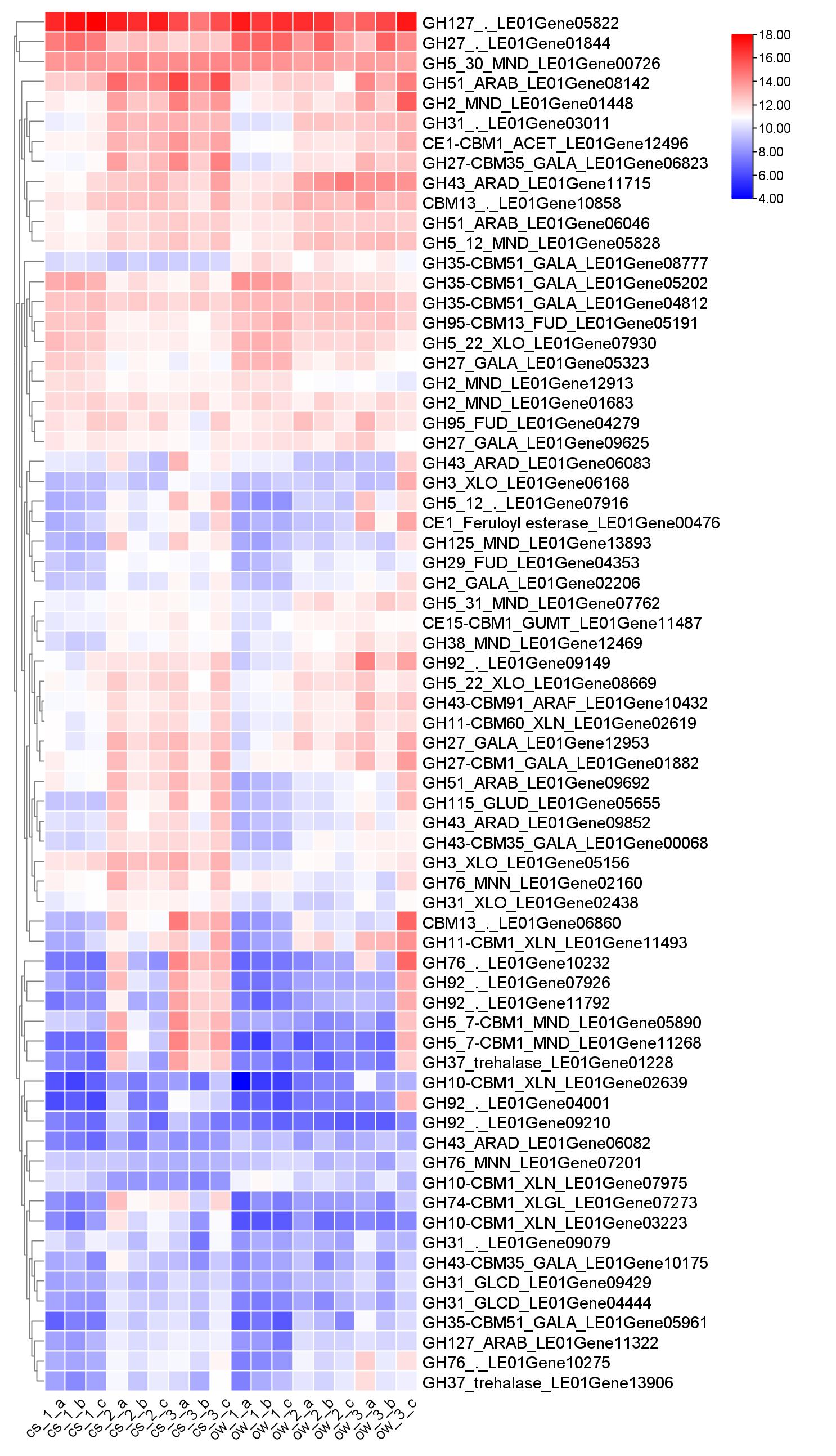


**Fig. S6. Expression heatmap of enzyme genes related to hemicellulose degradation during the spatial-temporal colonization using log2 (Normalized Count). Section locations for both substrates: 0–5 mm (section 1), ow_1 or cs_1; 15–20 mm (section 2), ow_2 or cs_2; 30–35 mm (section 3), ow_3 or cs_3.** Abbreviation: ACET, Acetylxylan esterase; ARAD, Arabinosidase; ARAF, Arabinofuranosidase; FUD, Fucosidase; GALA, Galactosidase; GLCD, Glucosidase; GLUD, Glucuronidase; GUMT, Glucuronoyl methylesterase; MND, Mannosidase; XLGL, Xyloglucanase; XLN, Xylanase; XLO, Xylosidase.


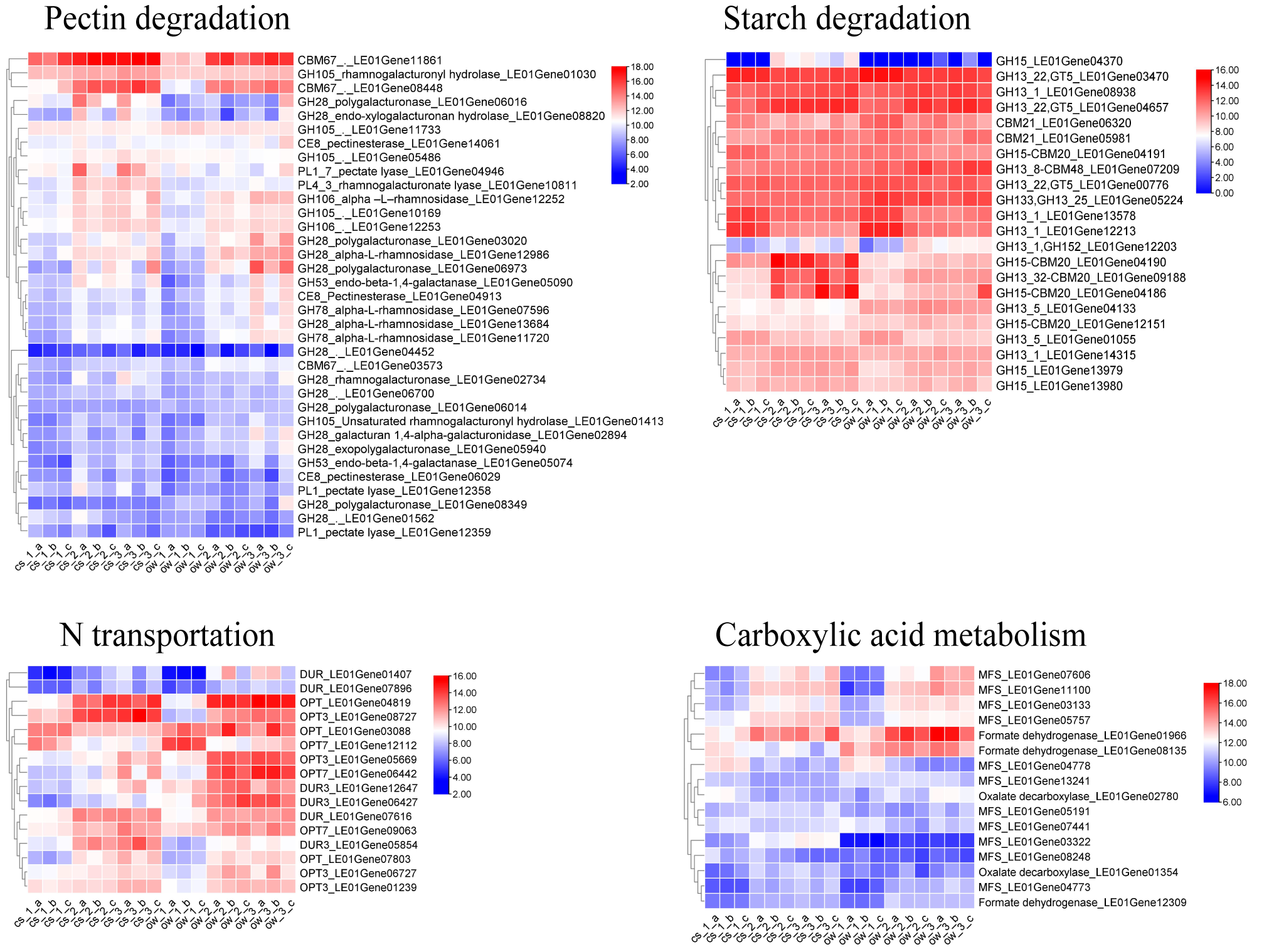


**Fig. S7.** **Expression heatmap of genes** **involved in carbohydrate and N metabolism using log2 (Normalized Count).** Section locations for both substrates: 0–5 mm (section 1), ow_1 or cs_1; 15–20 mm (section 2), ow_2 or cs_2; 30–35 mm (section 3), ow_3 or cs_3.


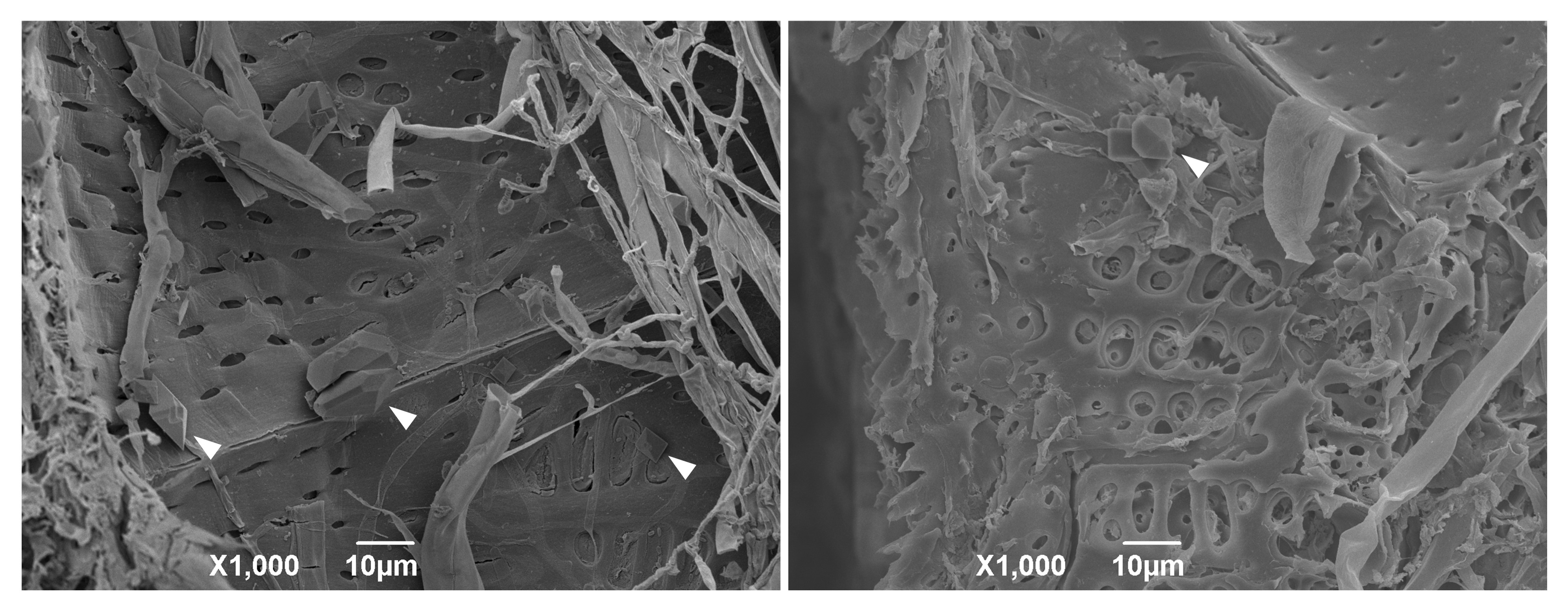


**Fig. S8. SEM observation of oxalate crystal deposited in oak wafer.** The white arrow indicated the oxalate crystal.

**Table S1. Primers used for qRT-PCR in this study**

| **Gene Name** | **Primer** | **Primer Sequences (5’ - 3’)** |
| --- | --- | --- |
| Actin | Leactin-RT-F | GGAGAAGATTTGGCATCACACA |
|  | Leactin-RT-R | GAAGAGCGAAACCCTCGTAGA |
| Endo-β-1,4-glucanase | LE01Gene04541-RT-F | CTGCCCCCACCGTTGCATCAGCCCT |
|  | LE01Gene04541-RT-R | TCAAAGTAGACGACTGATTACCCAC |
| Glucan 1,3-β-glucosidase | LE01Gene11039-RT-F | TCTCACTGGCTCCGCTTCAACCT |
|  | LE01Gene11039-RT-R | CAGCCGCTTCCGCCTCTTTCATA |
| Cellobiohydrolase I | LE01Gene04089-RT-F | TGGTCCTCGTTCTCAGTATTTGG |
|  | LE01Gene04089-RT-R | GCTGGAGGTAGTAGTGCCCGTA |
| Cellobiohydrolase I | LE01Gene04829-RT-F | TCTGGGACGATTACGCTGTGAA |
|  | LE01Gene04829-RT-R | CGTGGATGTGGCAGAGGAAGTA |
| Cellobiohydrolase II | LE01Gene10050-RT-F | AAGGTGATCCCAACTACGACGAG |
|  | LE01Gene10050-RT-R | TACCCAAACGATAGAATCAATCAAGG |
| Lytic polysaccharide monooxygenase | LE01Gene10266-RT-F | TCAACTCCAAGCGGACAATACC |
|  | LE01Gene10266-RT-R | CAGAATAAGCACCTGGGAAAGC |
| β-glucosidase | LE01Gene10512-RT-F | CTTCAGGCTCCGTCTCCTTTAT |
|  | LE01Gene10512-RT-R | ATCCCAGATGCTCATTTCTTTCAG |
| β-glucosidase | LE01Gene10634-RT-F | CTGCCGTCGTATGGAGTGGTT |
|  | LE01Gene10634-RT-R | TGTGCGGAATAATCGGTTGTG |
| Exo-1,4-β-xylosidase | LE01Gene05156-RT-F | GCACAATTACACCGACACGC |
|  | LE01Gene05156-RT-R | AAGTATCCGAGCCGAACGAG |
| Endo-1,4-β-xylanase | LE01Gene07975-RT-F | GATTCATTCAGTATTTCGCCTTGC |
|  | LE01Gene07975-RT-R | TCCCGAGTATGCTGGTTTCTTT |
| LACC 9 | LE01Gene04648-RT-F | GCATCCCTTCCACCTCCAT |
|  | LE01Gene04648-RT-R | CGGGCACAACTCAGTCCAG |
| LACC 12 | LE01Gene08149-RT-F | TGGATTGCGTGGAGCCTTTG |
|  | LE01Gene08149-RT-R | TCGGGACAGTACCGCTTTGG |
| Manganese peroxidase | LE01Gene09060-RT-F | CTGGACGACCGAATGCTACTGC |
|  | LE01Gene09060-RT-R | CTGCGAGTCGAAAGTGAAGGGA |

**References:**

Bolger AM, Marc L, Bjoern U (2014). Trimmomatic: a flexible trimmer for Illumina sequence data. *Bioinformatics (Oxford, England)* **30:** 2114–2120.

Chen C, Chen H, Zhang Y, Thomas HR, Frank MH, He Y *et al* (2020). TBtools: An Integrative Toolkit Developed for Interactive Analyses of Big Biological Data. *Molecular Plant* **13:** 1194–1202.

Kim D, Langmead B, Salzberg SL (2015). HISAT: A fast spliced aligner with low memory requirements. *Nature Methods* **12:** 357–360.

Langdon WB (2015). Performance of genetic programming optimised Bowtie2 on genome comparison and analytic testing (GCAT) benchmarks. *BioData Mining* **8:** 1.

Love MI, Huber W, Anders S (2014). Moderated estimation of fold change and dispersion for RNA-seq data with DESeq2. *Genome Biology* **15:** 550.

Tarazona S, Garcia-Alcalde F, Dopazo J, Ferrer A, Conesa A (2011). Differential expression in RNA-seq: A matter of depth. *Genome Research* **21:** 2213–2223.
